# Supplementary material for: Effects of mental health interventions for students in higher education are sustainable over time: a systematic review and meta-analysis of randomized controlled trials
Source: PeerJ. 2018 Apr 2;6:e4598. doi: 10.7717/peerj.4598 (PMC5885977; doi:10.7717/peerj.4598)
Supplement: Supplemental Information 1 — (A) Participants’ characteristics are reported as in the original trials. Several studies are missing the educational level or gender frequencies. (B) Comparator group defined in the original study as waitlist with delayed intervention was not included if intervention was provided before the end of follow-up Chase JA; (34). Comparison was made between the remaining trial arms. Table reported comparators used in meta-analysis. (C) In the trials with multiple interventions of similar nature, i.e. if interventions shared the same features and components and, therefore, belonged to the same category, effect sizes (Hedges’ g) calculated for each intervention were combined (35, 41). Similar approach was applied to studies with multiple control groups (35, 42, 43). An exception was a study by Kanji N et al. (38), where two control groups–an attention control and a time control–were included separately as considered to be different in approach and content and, thus, representing active and inactive comparisons, respectively. (D) As suggested by Conley, et al. (15), original interventions were grouped into: (i) cognitive behavior therapy-related if focusing on identifying and changing unhelpful cognitions, behaviors and emotional regulation; (ii) mind-body-related, i.e. interventions that facilitate the mind’s capacity to affect bodily function and symptoms; and (iii) psycho-educational-related focusing on information, discussion and didactic communication on, e.g. stress-reduction and coping. Categorization was based on the original definitions, if provided, and otherwise by us. (E) Adapted Schedule for Affective Disorders and Schizophrenia for School-Age Children (K-SADS); Anxiety Sensitivity Index (ASI); Beck Anxiety Inventory (BAI); Beck Depression Inventory (BDI); Beck & Srivastava Stress Inventory (BSSI); Center for Epidemiologic Studies Depression Scale (CES-D); Chinese Perceived Stress Scale (CPSS); Depression Anxiety Stress Scales (DASS-21); Fordyce Emotions Questio [file peerj-06-4598-s001.docx]

**Table S1. Characteristics of included studies**

| Study, Year, Country | Participants ^a^ | **Intervention (I)**  **Comparator (C) ^b, c^** | Intervention and comparator categories ^d^ | **Delivery / Format** | **Intervention length** | **Outcome measure (scale) ^e^** | **Outcome definition and category (Mental Ill Health (MIH) & Positive Mental Health (PMH) &Academic Performance) (AP))^f, g^** | **Post-int. follow-up ^h, i^** |
| --- | --- | --- | --- | --- | --- | --- | --- | --- |
| Braithwaite SR, 2009, US [52] | Undergraduate psychology students  18 M/ 51 F | I: Relationship educationC: Placebo38 I/ 39 C | Psycho-educational Inactive | Universal / Individual internet-based | 1 session+ emails,  ~7 weeks | BDI  BAI | Depressive symptoms (MIH)  Anxiety symptoms (MIH | 8 months |
| Chase JA, 2013, US **[34]** | Psychology majors 29 M/ 103 F | I1: Goal setting + valuesC: Goal setting alone 51 I/ 48 C | Mind-body related Active | Universal / Individual internet-based | 1 session+ emails + 2.5 h individual goal setting  ~4 weeks | GPA | Academic performance (AP) | 3 months |
| Cheng M, 2015, China **[53]^j^** | University students 30 M/ 36 F | I: Meaning-centered approach C: No intervention  34 I/ 32 C | Psycho-educational Inactive | Universal / Group | 9 sessions,2-2.5 h/each,9 weeks | RSES | Self-esteem (PMH) | 3 months |
| Chiauzzi E, 2008, US **[35]^k^** | 1^st^-4^th^-yr students with high stress perception  116 M/123 F | I: Stress management C1: Control website  C2: No treatment  77 I/ 78 C1/80 C2 | CBT-related  Inactive  Inactive | Selective / Individual internet-based | 4 sessions,  20 min/each,  2 weeks | HPLP SM | Stress management (PMH) | 5 months |
| Erogul M, 2014, US **[54]** | 1^st^-yr medical students  31 M/ 26 F | I: MBSR  C: No treatment  28 I / 29 C | Mind-body related  Inactive | Universal / Group | 8 sessions,  75 min/each,  8 weeks | PSS-10 SCS  RS | Perceived stress (MIH) Self-compassion (PMH)  Resilience (PMH) | 6 months |
| Fontana AM, 1999, US **[55]** | Undergraduate psychology students  22 M/ 14 F | I: Peer-led stress inoculation training C: Waitlist  18 I / 18 C | CBT-related  Inactive | Universal / Group | 6 sessions,  45 min**/**each | STAI | State anxiety (MIH) | 6 months |
| Franklin J, 2012, Australia  **[56]** | 1^st^-yr students  21 M/ 31 F (in intervention groups) | I1: PAAL I2: Self-regulation  C: No-treatment  27 I1/25 I2/2183C | Psycho-educational  Psycho-educational  Inactive | Universal / in pairs | 7 sessions,9 h in total, 7 weeks | GPA | Academic performance (AP) | 12 months  18 months |
| Gortner E-M, 2006, US **[57]** | Undergraduate students with elevated depressive symptoms in past and low current symptoms  27 M/ 70 F | I: Expressive writing  C: Control writing  57 I/ 40 C | Mind-body related  Inactive | Selective / Group | 3 sessions,  20 min**/**each,  3 days | BDI | Depressive symptoms (MIH) | 6 months |
| Hamdan-Mansour AM, 2009, Jordan **[58]** | Students with elevated depressive symptoms  46 M/ 38 F | I: Teaching Kids to Cope modified  C: No- treatment  44 I/ 40 C | CBT-related | Selective / Group | 10 sessions,  45 min/each,  10 weeks | PSS-10  BDI  WCQ  WCQ | Perceived stress (MIH)  Depressive symptoms (MIH)  Passive coping (MIH)  Active coping (PMH) | 3 months |
| Higgins D, 2006, US **[36]** | 1^st^-yr students with elevated symptoms of worry  25 M/ 53 F | I: CBT workshop C: No-treatment  38 I/ 40 C | CBT-related  Inactive | Selective / Group | 2 sessions,  2 h/each,  2 days | PSWQ  BDI  GADQ-IV  BAI | Self-reported worry (MIH)  Depressive symptoms (MIH)  Anxiety symptoms (MIH)  State anxiety (MIH) | 6 months  12 months |
| Jones MC, 2000, Scotland **[37]** | Nursing students with elevated symptoms of distress  11 M/ 68 F | I: Multi-modal stress management C: Waitlist  40 I/ 39 C | CBT-related | Selective / Group | 6 sessions,  2 h/each,  6 weeks | GHQ-30 STAI  STAI  BDI  BSSI  WCQ  WCQ  WCQ | Psychological distress (MIH)  State anxiety (MIH)  Trait anxiety (MIH)  Depressive symptoms (MIH)  Perceived stress (MIH)  Passive coping (MIH)  General coping (PMH)  Direct coping (PMH) | 3 months |
| Kanji N, 2006, UK **[38]** | Undergraduate nursing students 8 M/ 85 F | I: Autogenic training  C1: Attention control (laughter therapy)  C2: Time control  32 I/ 30 C1/ 31 C2 | Mind-body-related  Active  Inactive | Universal / Group | 8 sessions,  2 h/each,  8 weeks | STAI  STAI | State anxiety (MIH)Trait anxiety (MIH) | 3 months  6 months  9 months  12 months |
| Kattelmann K, 2014, US **[59]** | 1^st^-3^rd^-yr students  505 M/ 1134 F | I: Young Adults Eating and Active for Health C: No treatment  824 I/815 C | Psycho-educational  Inactive | Universal / Individual internet-based | 10 sessions+ emails,  10 weeks | PSS-14 | Perceived stress (MIH) | 12 months |
| Kenardy J, 2006, Australia **[60]** | 1^st^-yr psychology students with elevated symptoms of anxiety | I: Online Anxiety Prevention ProgramC: No treatment 36 I/ 38 C | CBT-related | Selective / Individual internet-based | 6 sessions, 6 weeks | ASI  CES-D | Anxiety symptoms (MIH) Depressive symptoms (MIH) | 4½ months |
| Li M, 2015, China **[61]** | 1^st^-2^nd^-yr students  36 M/ 170 F | I: Baduanjin exercises C: Physical activity as-usual  101 I/ 105 C | Mind-body related  Inactive | Universal, / Group | 60 sessions,  1 h/each,  12 weeks | PSQI  CPSS  GSES  SES | Quality of sleep (MIH)  Stress (MIH)  Self-efficacy (PMH)  Self-esteem (PMH) | 3 months |
| Mak WWS, 2015, Honkong **[41]** | University students and staff 95 M/ 191 F | I1: HAPA-enhanced mindfulness I2: Basis mindfulness  C: Waitlist  105 I1/104 I2/ 79 C | Mind-body related  Mind-body related  Inactive | Universal **/** Individual internet-based | 8 sessions,  30 min/each,  8 weeks | PSS-10 DASS-21  WHO-5 | Perceived stress (MIH)  Psychological distress (MIH)  Mental well-being (PMH) | 3 months |
| Pachankis JE, 2010, US **[62]** | Undergraduate students  77 M/ 0 F | I1: Expressive writing only I2: Writing & Reading  C: Neutral writing  27 I1/ 25 I2/25 C | Mind-body related  Mind-body related  Inactive | Selective / Individual | 3 sessions,  20 min/each,  3 days | CES-D  SCL-90  RSES | Depressive symptoms (MIH)  Psychological distress (MIH)  Self-esteem (PMH) | 3 months |
| Peden AR, 2001, US **[39]** | University students with elevated symptoms of depression  0 M/ 92 F | I: CBT C: No treatment  46 I/ 46 C | CBT-related  Inactive | Selective / Group | 6 sessions,  1 h/each,  6 weeks | CES-D BAI  RSES | Depressive symptoms (MIH) Anxiety symptoms (MIH) Self-esteem (PMH) | 6 months  18 months |
| Reavley NJ, 2014, Australia **[46]** | University students  293 M/ 653 F | I: MindWise C: No treatment  426 I/ 341 C | Psycho-educational | Universal / Group & individual internet-based | 2 academic years | K6 | Psychological distress (MIH) | 6 months  18 months |
| Rohde, 2014, US **[42]** | 1^st^ & 2^nd^-yr students with elevated symptoms of depression 25 M/ 57 F | I1: Brief CBT C1: Bibliotherapy  C2: Brochure  27 I/ 22 C1/ 33 C2 | CBT-related  Inactive  Inactive | Selective / Group | 6 sessions,  1 h/each,  6 weeks | K-SADS | Psychological distress (MIH) | 6 months  18 months |
| Seligman, 1999, US **[40]^l^** | 1^st^-yr students with elevated symptoms of depression 111 M/ 120 F | I: Prevention workshop C: No treatment 106 I/ 119 C | CBT-related  Inactive | Selective / Group | 8 sessions,  2 h /each,  8 weeks | BDI  HDRS  BAI  HARS | Depressive symptoms (MIH)  Depressive symptoms (MIH)  Anxiety symptoms (MIH)  Anxiety symptoms (MIH) | 6 months  12 months  18 months |
| Seligman MEP,  2007, US **[44]** | 1^st^-yr students with elevated symptoms of depression 79 M/ 148 F | I: Workshop C: No treatment  102 I/ 125 C | CBT-related  Inactive | Selective / Group | 8 sessions + homework,  2 h /each,  8 weeks | BDI  BAI  Fordyce Happy | Depressive symptoms (MIH)Anxiety symptoms (MIH) Happiness (PMH) | 4 months  6 months |
| Shapiro SL, 2011, US **[63]** | 1^st^-3^rd^-yr students  4 M/26 F | I: MBSR C: Waitlist  15 I/ 15 C | Mind-body-related  Inactive | Universal / Group | 8 sessions, 90 min/each,  8 weeks | PSS-10  SCS  SWB score^m^ | Stress (MIH) Self-compassion (PMH)  Subjective well-being (PMH) | 12 months |
| Vazquez F, 2012, Spain **[45]** | University students with elevated symptoms of depression  24 M/ 109 F | I: CBT C: Relaxation training  70 I/ 63 C | Mind-body-related  Active | Selective / Group | 8 sessions + homework, 90 min/each, 8 weeks | CES-D  BAI | Depressive symptoms (MIH)  Anxiety symptoms (MIH) | 3 months  6 months |
| Yang W, 2014,  China **[43]** | 2^nd^-yr students with elevated symptoms of depression  22 M/ 55 F | I: Attention bias modificationC1: Placebo C2: No treatment  27 I/27 C1/ 23 C2 | CBT-related  Inactive  Inactive | Selective / Group & individual internet-based | 8 sessions,  12 min/each,  2 weeks | BDI-II STAI-T | Depressive symptoms (MIH)  Trait anxiety (MIH) | 3 months  7 months |
| Zheng G, 2015, China **[64]** | 1^st^ & 2^nd^-yr students  65 M/ 133 F | I: Tai-Chi Chuang ExerciseC: Physical activity as usual 95 I/ 103 C | Mind-body related  Inactive | Universal / Group | 60 sessions,  60 min/each, 12 weeks | PSQI  CPSS  SES  GSES | Quality of sleep (MIH)  Stress (MIH)  Self-esteem (PMH)  Self-efficacy (PMH) | 3 months |

^a^ Participants’ characteristics are reported as in the original trials. Several studies are missing the educational level or gender frequencies.

^b^ Comparator group defined in the original study as waitlist with delayed intervention was not included if intervention was provided before the end of follow-up Chase JA; (34). Comparison was made between the remaining trial arms. Table reported comparators used in meta-analysis.

^c^ In the trials with multiple interventions of similar nature, i.e. if interventions shared the same features and components and, therefore, belonged to the same category, effect sizes (Hedges’ g) calculated for each intervention were combined (35, 41). Similar approach was applied to studies with multiple control groups (35, 42, 43). An exception was a study by Kanji N et al. (38), where two control groups – an attention control and a time control – were included separately as considered to be different in approach and content and, thus, representing active and inactive comparisons, respectively.

^d^ As suggested by Conley, et al. (15), original interventions were grouped into: (i) cognitive behaviour therapy-related if focusing on identifying and changing unhelpful cognitions, behaviours and emotional regulation; (ii) mind-body-related, i.e. interventions that facilitate the mind's capacity to affect bodily function and symptoms; and (iii) psycho-educational-related focusing on information, discussion and didactic communication on, e.g. stress-reduction and coping. Categorization was based on the original definitions, if provided, and otherwise by us.

^e^

Adapted Schedule for Affective Disorders and Schizophrenia for School-Age Children (K-SADS); Anxiety Sensitivity Index (ASI); Beck Anxiety Inventory (BAI); Beck Depression Inventory (BDI); Beck & Srivastava Stress Inventory (BSSI); Center for Epidemiologic Studies Depression Scale (CES-D); Chinese Perceived Stress Scale (CPSS); Depression Anxiety Stress Scales (DASS-21); Fordyce Emotions Questionnaire (Fordyce Happy); Generalized Anxiety Disorder Questionnaire (GADQ-IV); General Health Questionnaire); General Self-efficacy Scale (GSES); Grade Point Average (GPA); Hamilton Anxiety Rating Scale (HARS); Hamilton Depression Rating Scale (HDRS); Health Action Process Approach **(**HAPA**);** Health Promoting Lifestyle Profile (HPLP); Kessler Psychological Distress Scale, 6 items (K6); Mindfulness based stress reduction (MBSR); Overall Subjective Wellbeing score (SWB score); Penn Scale Worry questionnaire (PSWQ); Perceived Stress Scale; Pittsburgh Sleep Quality Index (PSQI); Preparation, Action, Adaptive Learning (PAAL); Resilience Scale (RS); Rosenberg Self Esteem Scale (RSES); Self-Compassion Scale (SCS); Self Esteem Scale (SES) = Chinese version; Self-Symptom Intensity Scale (SCL-90); State and Trait Anxiety Instrument (STAI); State-Trait Anxiety Inventory-Trait version (STAI-T).The Ways of Coping Questionnaire (WCQ); World Health Organization Well-Being Index (WHO-5).

^f^ Outcomes were classified into mental ill-health: anxiety symptoms, depressive symptoms, psychological distress, (perceived) stress, self-reported worry, passive coping, (including suppression), and deteriorated quality of sleep. Further, positive mental health and academic performance outcomes: self-esteem, self-compassion, self-efficacy, mental or subjective well-being, resilience, active coping, (including general and direct coping), happiness, stress management and academic performance.

^g^ If the trial reported several measures for the same outcome at a given follow-up time point (e.g. for depressive symptoms assessed by both the Hamilton Depression Rating Scale and Beck Depression Inventory) the effect sizes (Hedges’ g) were combined to obtain the single outcome measure per intervention at each measurement point (36-40). That also refers to the state and trait anxiety (or anxiety symptoms) to be measured separately within the same study Higgins DM (36)), Jones MC (37), Kanji N (38). Likewise, measures of general and direct coping were combined within a study to obtain a single measure of active coping Jones MC (38).

^h^ If original study reported post-baseline follow-up it was re-calculated into post-interventional follow-up by subtracting the length of interventions from the reported months.

^i^ For meta-analysis post-intervention follow-ups were categorized as 3-6 months, 7-12 months, and 13-18 months. If a given study provided several outcome measures falling in the same length category (e.g., for both 3 and 6 month follow-ups), follow-up close to the upper boundary (i.e., 6 months) was chosen (38, 44, 45).

^j^ Depression and anxiety, both measured with GHQ-20, were not retrieved from Cheng M et al. (53) to be used in the meta-analysis due to unclearness with interpretation of original measurements.

^k^ Measures on anxiety from Chiauzzi E et al. (35) were not retrieved due to missing quantitative data.

^l^ Outcome measures during the follow-up periods longer than 18 months were not retrieved from Seligman et al. (1999) (47).

^m^ Overall Subjective Wellbeing score composed of scores measured by Positive and Negative Affect Schedule and The Satisfaction with Life Scale
