# Supplementary material for: Effects of mental health interventions for students in higher education are sustainable over time: a systematic review and meta-analysis of randomized controlled trials
Source: PeerJ. 2018 Apr 2;6:e4598. doi: 10.7717/peerj.4598 (PMC5885977; doi:10.7717/peerj.4598)
Supplement: Supplemental Information 2 — a Ratings are conducted according to the EPHPP Dictionary Guidelines and the EPHPP Quality Assessment Tool. The Global rating is compiled of: Strong (no weak component ratings), Moderate (one weak component rating), and Weak (two or more weak component ratings). [file peerj-06-4598-s002.docx]

**Table S2.**

**Study quality assessment by the tool Effective Public Health Practice Project (EPHPP)^a^**

| **Study - First author, Year,**  **Reference** | **Intervention with at least one mental ill health outcome** | **Intervention with at least one positive mental health outcome, including academic performance** | **Selection bias** | **Study design** | **Confounders** | **Blinding** | **Data collection method** | **Withdrawals and dropouts** | **Global rating** |
| --- | --- | --- | --- | --- | --- | --- | --- | --- | --- |
| Braithwaite SR, 2009 **[52]** | Yes | ̶ | Weak | Strong | Strong | Moderate | Strong | Moderate | Moderate |
| Chase JA, 2013 **[34]** | ̶ | Yes | Weak | Strong | Strong | Moderate | Strong | Moderate | Moderate |
| Cheng M, 2015 **[53]** | Yes | Yes | Weak | Strong | Weak | Moderate | Strong | Weak | Weak |
| Chiauzzi E, 2008 **[35]** | Yes | Yes | Moderate | Strong | Strong | Moderate | Strong | Strong | Strong |
| Erogul M, 2014 **[54]** | Yes | Yes | Moderate | Strong | Strong | Moderate | Strong | Moderate | Strong |
| Fontana AM, 1999 **[55]** | Yes | ̶ | Weak | Strong | Strong | Moderate | Strong | Moderate | Moderate |
| Franklin J, 2012 **[56]** | ̶ | Yes | Weak | Strong | Weak | Strong | Moderate | Strong | Weak |
| Gortner EM, 2006 **[57]** | Yes | ̶ | Weak | Strong | Weak | Moderate | Strong | Strong | Weak |
| Hamdan-Mansour AM, 2009 **[58]** | Yes | Yes | Weak | Strong | Strong | Moderate | Strong | Strong | Moderate |
| Higgins DM, 2006 **[36]** | Yes | ̶ | Weak | Strong | Strong | Moderate | Strong | Moderate | Moderate |
| Jones MC, 2000 **[37]** | Yes | Yes | Weak | Strong | Strong | Moderate | Strong | Strong | Moderate |
| Kanji N, 2006 **[38]** | Yes | ̶ | Weak | Strong | Weak | Weak | Strong | Weak | Weak |
| Kattelman KK, 2014 **[59]** | Yes | ̶ | Weak | Strong | Strong | Moderate | Strong | Weak | Weak |
| Kenardy J, 2006 **[60]** | Yes | ̶ | Weak | Strong | Strong | Moderate | Strong | Weak | Weak |
| Li M, 2015 **[61]** | Yes | Yes | Weak | Strong | Strong | Moderate | Strong | Strong | Moderate |
| Mak WWS, 2015 **[41]** | Yes | Yes | Moderate | Strong | Weak | Strong | Strong | Weak | Weak |
| Pachankis JE, 2010 **[62]** | Yes | Yes | Weak | Strong | Weak | Moderate | Strong | Weak | Weak |
| Peden AR, 2010 **[39]** | Yes | Yes | Weak | Strong | Strong | Moderate | Strong | Weak | Weak |
| Reavley NJ, 2014 **[46]** | Yes | ̶ | Weak | Strong | Strong | Moderate | Strong | Weak | Weak |
| Rohde P, 2014 **[42]** | Yes | ̶ | Weak | Strong | Strong | Moderate | Strong | Strong | Moderate |
| Seligman MEP, 1999 **[40]** | Yes | ̶ | Strong | Strong | Strong | Strong | Strong | Strong | Strong |
| Seligman MEP, 2007 **[44]** | Yes | Yes | Weak | Strong | Strong | Strong | Strong | Strong | Moderate |
| Shapiro SL, 2011 **[63]** | Yes | Yes | Moderate | Strong | Strong | Moderate | Strong | Strong | Strong |
| Vazquez FL, 2012 **[45]** | Yes | ̶ | Weak | Strong | Strong | Moderate | Strong | Strong | Moderate |
| Yang WY, 2014 **[43]** | Yes | ̶ | Weak | Strong | Moderate | Strong | Strong | Moderate | Moderate |
| Zheng G, 2015 **[64]** | Yes | Yes | Weak | Strong | Strong | Moderate | Strong | Strong | Moderate |

**^a^** Ratings are conducted according to the EPHPP Dictionary Guidelines and the EPHPP Quality Assessment Tool. The Global rating is compiled of: Strong (no weak component ratings), Moderate (one weak component rating), and Weak (two or more weak component ratings).
