# Supplementary material for: Effects of mental health interventions for students in higher education are sustainable over time: a systematic review and meta-analysis of randomized controlled trials
Source: PeerJ. 2018 Apr 2;6:e4598. doi: 10.7717/peerj.4598 (PMC5885977; doi:10.7717/peerj.4598)
Supplement: Supplemental Information 3 — k; number of studies (*)p < 0.1 *p < 0.05, **p < 0.01, ***p < 0.001. [file peerj-06-4598-s003.docx]

**Table S3 Meta-analysis for the specific mental ill health outcomes stratified by the length of post interventional follow-up periods.**

| **Outcomes** | **Length of post intervention follow-up periods (months)** | | |
| --- | --- | --- | --- |
|  | **3-6** | **7-12** | **13-18** |
| **Depressive symptoms (k)** | 12 | 4 | 2 |
| Hedges’ g (95% CI) | -0.33 (-0.58, -0.08) | -0.47 (-0.90, -0.04) | -0.30 (-0.51, -0.08) |
| Q (p-value)/ I^2^ | 54.54*** / 79.8% | 14.61** / 79.5% | 0.28 / 0.0% |
| **Anxiety symptoms (k)** | 10 | 6 | 1 |
| Hedges’ g (95% CI) | -0.31 (-0.51, -0.12) | -0.27 (-0.54, -0.01) | -0.32 (-0.59, -0.06) |
| Q (p-value)/ I^2^ | 29.04** / 69.0% | 17.97** / 72.2% | --- |
| **Symptoms of stress (k)** | 6 | 2 | 0 |
| Hedges’ g (95% CI) | -0.30 (-0.58, -0.03) | -0.08 (-0.19, 0.04) | --- |
| Q (p-value)/ I^2^ | 21.40** / 76.6% | 0.25 / 0.0% | --- |
| **Psychological distress (k)** | 5 | 1 | 1 |
| Hedges’ g (95% CI) | -0.16 (-0.43, 0.10) | 0.28 (-0.09, 0.65) | -0.02 (-0.16, 0.12) |
| Q (p-value)/ I^2^ | 25.68*** / 84.4% | --- | --- |
| **Self-reported worry (k)** | 1 | 1 | 0 |
| Hedges’ g (95% CI) | 0.23 (-0.29, 0.75) | 0.27 (-0.30, 0.84) | --- |
| Q (p-value)/ I^2^ | --- | --- | --- |
| **Quality of sleep (k)** | 2 | 0 | 0 |
| Hedges’ g (95% CI) | -0.10 (-0.29, 0.09) | --- | --- |
| Q (p-value)/ I^2^ | 0.09 / 0.0% | --- | --- |
| **Passive coping (k)** | 1 | 0 | 0 |
| Hedges’ g (95% CI) | -0.99 (-1.44, -0.54) | --- | --- |
| Q (p-value)/ I^2^ | --- | --- | --- |

k; number of studies

(*) p<0.1 * p<0.05, ** p< 0.01, ***p<0.001
