# Supplementary material for: Effects of mental health interventions for students in higher education are sustainable over time: a systematic review and meta-analysis of randomized controlled trials
Source: PeerJ. 2018 Apr 2;6:e4598. doi: 10.7717/peerj.4598 (PMC5885977; doi:10.7717/peerj.4598)
Supplement: Supplemental Information 4 — k; number of studies (*)p < 0.1 *p < 0.05, **p < 0.01, ***p < 0.001. [file peerj-06-4598-s004.docx]

**Table S4 Meta-analysis for the specific positive mental health and academic performance outcomes stratified by the length of post interventional follow-up periods.**

| **Outcomes** | **Length of post intervention follow-up periods (months)** | | |
| --- | --- | --- | --- |
|  | **3-6** | **7-12** | **13-18** |
| **Self-esteem (k)** | 5 | 0 | 1 |
| Hedges’ g (95% CI) | 0.41 (-0.05, 0.88) | --- | 0.29 (-0.29, 0.87) |
| Q (p-value)/ I^2^ | 29.50*** / 86.4% | --- | --- |
| **Academic performance (k)** | 1 | 1 | 1 |
| Hedges’ g (95% CI) | -0.11 (-0.50, 0.28) | 0.34 (-0.14, 0.82) | 0.37 (-0.17, 0.90) |
| Q (p-value)/ I^2^ | --- | --- | --- |
| **Self-efficacy (k)** | 2 | 0 | 0 |
| Hedges’ g (95% CI) | -0.08 (-0.40, 0.23) | --- | --- |
| Q (p-value)/ I^2^ | 2.65 / 62.2% | --- | --- |
| **Self-compassion (k)** | 1 | 1 | 0 |
| Hedges’ g (95% CI) | 1.04 (0.50, 1.59) | 0.35 (-0.35, 1.05) | --- |
| Q (p-value)/ I^2^ | --- | --- | --- |
| **Mental or subjective well-being (k)** | 1 | 1 | 0 |
| Hedges’ g (95% CI) | -0.31 (-0.52, -0.10) | 0.37 (-0.33, 1.07) | --- |
| Q (p-value)/ I^2^ | --- | --- | --- |
| **Resilience (k)** | 1 | 0 | 0 |
| Hedges’ g (95% CI) | 0.44 (-0.77, 0.96) | --- | --- |
| Q (p-value)/ I^2^ | --- | --- | --- |
| **Stress management (k)** | 1 | 0 | 0 |
| Hedges’ g (95% CI) | 0.00 (-0.20, 0.20) | --- | --- |
| Q (p-value)/ I^2^ | --- | --- | --- |
| **Active coping (k)** | 2 | 0 | 0 |
| Hedges’ g (95% CI) | 0.75 (0.19, 1.30) | --- | --- |
| Q (p-value)/ I^2^ | 1.39 / 27.9% | --- | --- |
| **Happiness rating (k)** | 1 | 0 | 0 |
| Hedges’ g (95% CI) | 0.27 (0.00, 0.53) | --- | --- |
| Q (p-value)/ I^2^ | --- | --- | --- |

k; number of studies

(*) p<0.1 * p<0.05, ** p< 0.01, ***p<0.001
