# Supplementary material for: Effects of mental health interventions for students in higher education are sustainable over time: a systematic review and meta-analysis of randomized controlled trials
Source: PeerJ. 2018 Apr 2;6:e4598. doi: 10.7717/peerj.4598 (PMC5885977; doi:10.7717/peerj.4598)

(A) Negative outcomes with 3-6 months of follow-up

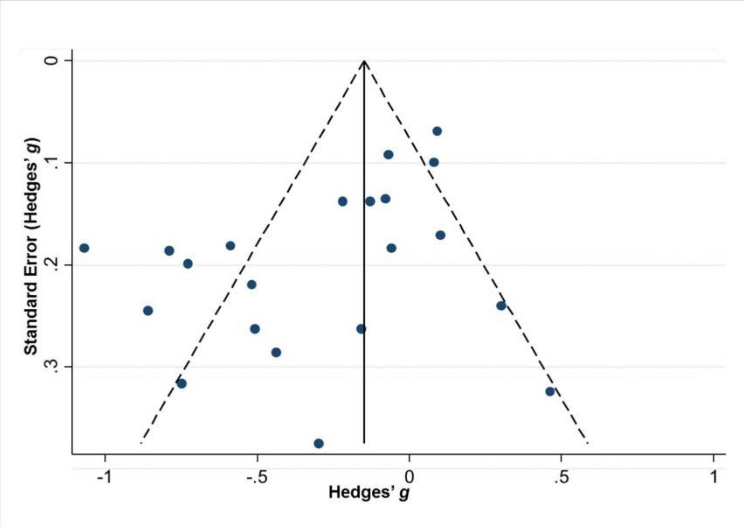

(B) Negative outcomes with 7-12 months of follow-up

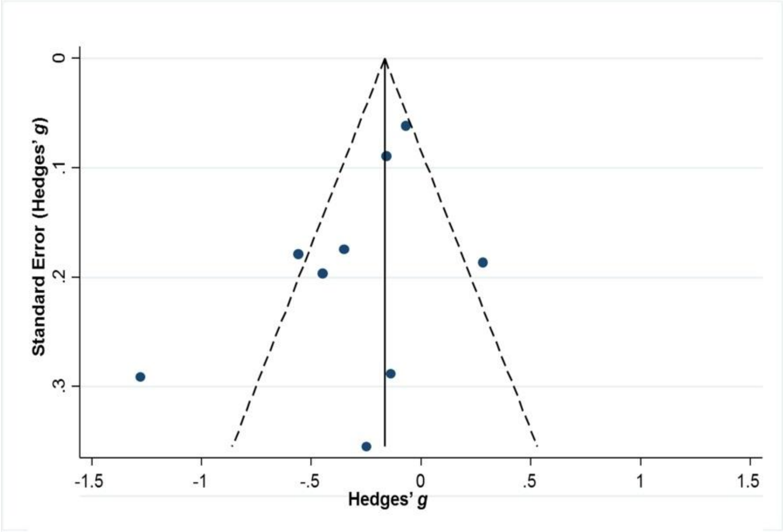

(C) Negative outcomes with 13-8 months of follow-up

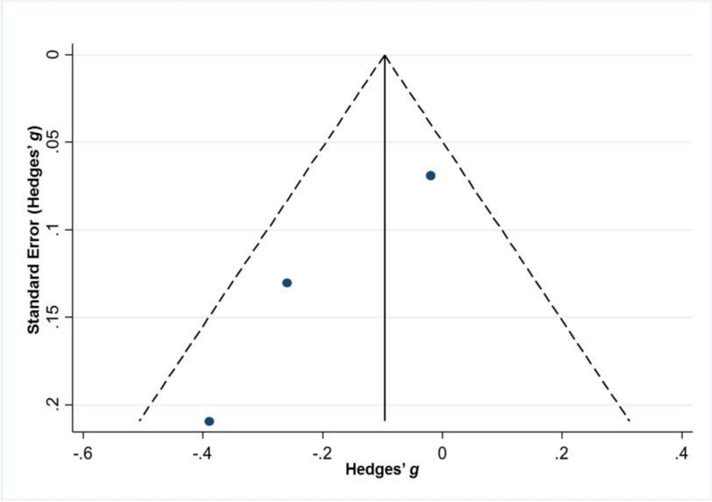

(D) Positive outcomes with 3-6 months of follow-up

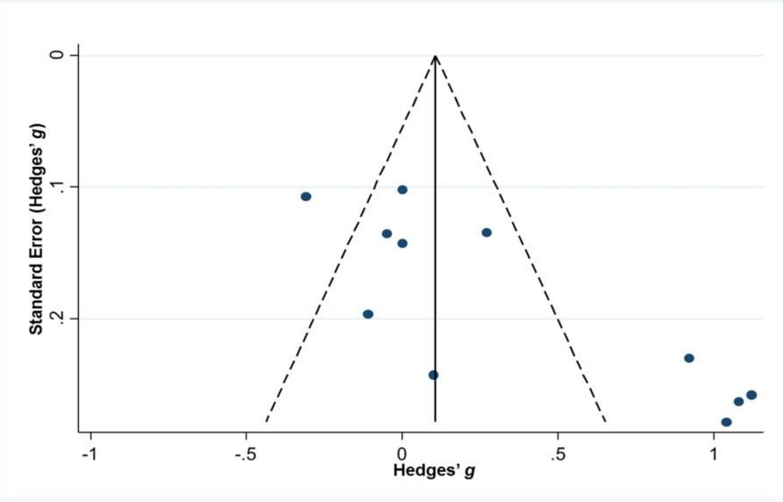

Supplement: Supplemental Information 6 — Funnel plots of standard error by Hedges’ g effect sizes for mental ill health and positive mental health and academic performance outcomes (hierarchically selected) from the trials with different lengths of post-intervention follow-ups: (A) Mental ill health outcomes with 3–6 months of follow-up; (B) Mental ill health outcomes with 7–12 months of follow-up; (C) Mental ill health outcomes with 13–18 months of follow-up; (D) Positive mental health and academic performance outcomes with 3–6 months of follow-up. No funnel plots were built for positive mental health and academic performance outcomes with 7–12 months and 13–18 months of follow-up due to lack of original data (only two studies were included in each corresponding category). [file peerj-06-4598-s006.pdf]
