# Supplementary material for: Effects of mental health interventions for students in higher education are sustainable over time: a systematic review and meta-analysis of randomized controlled trials
Source: PeerJ. 2018 Apr 2;6:e4598. doi: 10.7717/peerj.4598 (PMC5885977; doi:10.7717/peerj.4598)
Supplement: Supplemental Information 7 — Regina Winzer, Lene Lindberg, Karin Guldbrandsson, Anna Sidorchuk. Are effects of mental health promoting interventions among students in tertiary educational settings sustainable? A systematic review. PROSPERO 2015:CRD42015029353 Available from http://www.crd.york.ac.uk/PROSPERO_REBRANDING/display_record.asp?ID=CRD42015029353. [file peerj-06-4598-s007.pdf]

PROSPERO International prospective register of systematic reviews

Are effects of mental health promoting interventions among students in tertiary educational settings sustainable? A systematic review

Regina Winzer, Lene Lindberg, Karin Guldbrandsson, Anna Sidorchuk

Citation

Regina Winzer, Lene Lindberg, Karin Guldbrandsson, Anna Sidorchuk. Are effects of mental health promoting interventions among students in tertiary educational settings sustainable? A systematic review. PROSPERO 2015:CRD42015029353 Available from
http://www.crd.york.ac.uk/PROSPERO_REBRANDING/display_record.asp?ID=CRD42015029353

Review question(s)

Do interventions on individual-, group- or structural level promote mental health or prevent mental ill-health among students in tertiary educational settings compared to students who have not been provided such interventions?

Searches

Data-bases preliminary searched from January 1, 1995 - May 22, 2015

• MEDLINE (Ovid)

• PsycInfo (Ovid)

• Eric (Ovid)

• Scopus

• Dissertations and Theses (ProQuest) for gray literature

No language restrictions were applied during the search process, but to be included into the review, only publications in English language will be eligible. These data-bases will be completed in December 2015/ January 2016 and OpenGrey and GreyNet will be searched up to December 31, 2015. Reference lists of eligible studies will be hand-searched.

Types of study to be included

RCT:s published in peer-reviewed journals only. Follow-up > 3 months.

Condition or domain being studied

Mental health, mental ill-health, campus- and student health services settings.

Participants/ population

Students in tertiary educations with or without self-rated mental ill-health in university or college settings.

Intervention(s), exposure(s)

Mental health promoting and mental ill-health preventing interventions, universal or selective, on organizational, group-based or individual level, web-or computer-based.

Comparator(s)/ control

No intervention.

Context

University-/campus settings.

Outcome(s)

Primary outcomes

Mental health and mental ill-health, well-being, coping, locus of control, study achievement/performance, resilience, self-esteem/-compassion, symptoms of anxiety, symptoms of depression, psychological distress, worry, fatigue, sleeping problems, stress management, perceived stress.

Secondary outcomes

None

Risk of bias (quality) assessment

Risk of bias and the quality within the original studies will be assessed by using the Effective Public Health Practise Tool (EPHPP). The implementation quality of the included studies will be assessed by the Quality Implementation Framework and the PRISMA standard will guide the reporting of the review.

Strategy for data synthesis

The data will be reported on an aggregated level. If our data prove to be sufficiently homogenous, a meta-analysis will be provided for applicable outcomes. In other case, we will prepare a narrative synthesis.

Analysis of subgroups or subsets

None planned.

Dissemination plans

We have ongoing consultations with representatives and different professionals from Student health services in Sweden. Our review could be used by them as a guide.

Contact details for further information

Miss Winzer

Public Health Agency of Sweden

SE-171 82 Solna, Sweden

& Karolinska Institutet, Department of Public Health Sciences, SE-171 77 Stockholm, Sweden

regina.winzer@folkhalsomyndigheten.se

Organisational affiliation of the review

Public Health Agency of Sweden & Karolinska Institutet

www.folkhalsomyndigheten.se & www.ki.se

Review team

Miss Regina Winzer, Public Health Agency of Sweden & Karolinska Institutet
Dr Lene Lindberg, Karonlinska Institutet
Dr Karin Guldbrandsson, Public Health Agency of Sweden & Karolinska Institutet
Dr Anna Sidorchuk, Karolinska Institutet

Collaborators

Mr Carl Gornitzki, Karolinska Institutet, University Library
Mr Anders Wändahl, Karolinska Institutet, University Library
Dr Anna Månsdotter, Pubic Health Agency of Sweden & Karolinska Institutet
Mrs Annika Frykholm, Pubic Health Agency of Sweden
Dr Sven Bremberg, Public Health Agency of Sweden & Karolinska Institutet

Anticipated or actual start date

22 May 2015

Anticipated completion date
16 September 2024

Funding sources/sponsors
The systematic review is conducted in accordance with a governmental assignment and is funded by the Public Health Agency of Sweden.

Conflicts of interest
None known

Language
English

Country
Sweden

Subject index terms status
Subject indexing assigned by CRD

Subject index terms
Humans; Mental Health; Students

Stage of review
Ongoing

Date of registration in PROSPERO
25 November 2015

Date of publication of this revision
12 May 2016

Stage of review at time of this submission	Started	Completed
Preliminary searches	Yes	No
Piloting of the study selection process	Yes	No
Formal screening of search results against eligibility criteria	Yes	No
Data extraction	Yes	No
Risk of bias (quality) assessment	Yes	No
Data analysis	Yes	No

PROSPERO
International prospective register of systematic reviews
The information in this record has been provided by the named contact for this review. CRD has accepted this information in good faith and registered the review in PROSPERO. CRD bears no responsibility or liability for the content of this registration record, any associated files or external websites.
