# Supplementary material for: Effects of mental health interventions for students in higher education are sustainable over time: a systematic review and meta-analysis of randomized controlled trials
Source: PeerJ. 2018 Apr 2;6:e4598. doi: 10.7717/peerj.4598 (PMC5885977; doi:10.7717/peerj.4598)
Supplement: Supplemental Information 8 [file peerj-06-4598-s008.docx]

**Data S2 Documentation of search strategies for Medline, PsycInfo, Eric, and Scopus.**

Documentation of search strategies

Research question: Are interventions among students in tertiary education sustainable over time?

Date: March–May 2015

Name of researcher: Regina Winzer, The Public Health Agency of Sweden & Karolinska Institutet

Librarians: Anders Wändahl & Carl Gornitzki, University Library, Karolinska Institutet

Limits to publication year: Jan 1, 1995 – May 21, 2015

Total number of hits:

- Before deduplication: 6,004
- After deduplication: 5,016

Databases:

- Medline (Ovid)
- PsycInfo (Ovid)
- Eric (Ovid)
- Scopus

1. Medline (Ovid)

Date of search: May 21, 2015

Number of hits: 961

Updated: December 1, 2016. See below.

RCT-filter: Cochrane Highly Sensitive Search Strategy for identifying randomized trials in MEDLINE: sensitivity- and precision-maximizing version (2008 revision); Ovid format: <http://handbook.cochrane.org/chapter_6/box_6.4.d_cochrane_hsss_2008_sensprec_ovid.htm>

| 1. exp Student Health Services/  2. exp universities/ and exp students/  3. ((student or campus or university or college) adj3 health adj3 (clinic* or service* or unit* or counsel* or center*)).tw.  4. ((student* or undergrad* or sophom* or master*) adj3 (higher or college* or universit* or tertiar*)).tw.  5. or/1-4  6. exp Mental Disorders/  7. exp Mental Health/  8. exp Psychiatry/  9. exp Psychology/  10. (anxi* or depress* or psych* or mental* or stress* or emotion* or nervous* or hypervigil* or sleep* or mood* or well-being or wellbeing or Wellness or resilience or coping or cope or competenc* or study achievement* or self-esteem or self-efficacy).tw.  11. or/6-10  12. exp Health Promotion/  13. exp Counseling/  14. (prevent* or promot* or improv* or intervent* or favour* or favor* or counsel* or councel* or avoid* or inhibit* or program* or support* or skill*).tw.  15. or/12-14  16. 5 and 11 and 15  17. randomized controlled trial.pt.  18. controlled clinical trial.pt.  19. randomi?ed.ab.  20. placebo.ab.  21. clinical trials as topic.sh.  22. randomly.ab.  23. trial.ti.  24. 17 or 18 or 19 or 20 or 21 or 22 or 23  25. 16 and 24  26. limit 16 to "reviews (best balance of sensitivity and specificity)"  27. 25 or 26 |
| --- |

2. PsycInfo (Ovid)

Date: May 21, 2015

Number of hits: 2,462

Updated: December 1, 2016. See below.

RCT-filter:

Eady AM, Wilczynski NL, Haynes RB. PsycINFO search strategies identified methodologically sound therapy studies and review articles for use by clinicians and researchers. J Clin Epidemiol. 2008 Jan;61(1):34-40. <http://www.ncbi.nlm.nih.gov/pubmed/?term=18083460>

| 1. exp Student Personnel Services/  2. exp Colleges/  3. exp College Students/  4. 2 or 3  5. exp Health Care Services/  6. 4 and 5  7. ((student or campus or university or college) adj3 health adj3 (clinic* or service* or unit* or counsel* or center*)).tw.  8. ((student* or undergrad* or sophom* or master*) adj3 (higher or college* or universit* or tertiar*)).tw.  9. 1 or 6 or 7 or 8  10. exp Mental disorders/  11. exp Mental Health/  12. exp Psychiatry/  13. exp Psychology/  14. (anxi* or depress* or psych* or mental* or stress* or emotion* or nervous* or hypervigil* or sleep* or mood* or well-being or wellbeing or wellness or resilience or coping or cope or competenc* or study achievement* or self-esteem or self-efficacy).tw.  15. or/10-14  16. exp Health Promotion/  17. exp Counseling/  18. (prevent* or promot* or improv* or intervent* or favour* or favor* or counsel* or councel* or avoid* or inhibit* or program* or support* or skill*).tw.  19. or/16-18  20. 9 and 15 and 19  21. double-blind.tw.  22. random* assigned.tw.  23. control.tw.  24. 21 or 22 or 23  25. 20 and 24  26. review.ti.  27. 20 and 26  28. 25 or 27 |
| --- |

3. Eric (Ovid)

Date: May 22, 2015

Number of hits: 420

Updated: December 1, 2016. See below.

RCT-filter as in PsycInfo

| 1. exp Student Personnel Services/  2. exp Colleges/  3. exp College Students/  4. 2 or 3  5. exp Health Services/  6. 4 and 5  7. ((student or campus or university or college) adj3 health adj3 (clinic* or service* or unit* or counsel* or center*)).ti,ab.  8. ((student* or undergrad* or sophom* or master*) adj3 (higher or college* or universit* or tertiar*)).ti,ab.  9. 1 or 6 or 7 or 8  10. exp Mental Disorders/  11. exp Mental Health/  12. exp Psychiatry/  13. exp Psychology/  14. (anxi* or depress* or psych* or mental* or stress* or emotion* or nervous* or hypervigil* or sleep* or mood* or well-being or wellbeing or wellness or resilience or coping or cope or competenc* or study achievement* or self-esteem or self-efficacy).ti,ab.  15. 10 or 11 or 12 or 13 or 14  16. exp Health Promotion/  17. exp Counseling/  18. (prevent* or promot* or improv* or intervent* or favour* or favor* or counsel* or councel* or avoid* or inhibit* or program* or support* or skill*).ti,ab.  19. 16 or 17 or 18  20. 9 and 15 and 19  21. double-blind.ti,ab.  22. random* assigned.ti,ab.  23. control.ti,ab.  24. 21 or 22 or 23  25. 20 and 24  26. review.ti.  27. 20 and 26  28. 25 or 27 |
| --- |

4. Scopus

Date: May 22, 2015

Number of hits: 2,161

Updated: December 1, 2016. See below.

RCT-filter as in PsycInfo

| #1 = TITLE-ABS((student OR campus OR university OR college) W/3 health W/3 (clinic* OR service* OR unit* OR counsel* OR center*))  #2 = TITLE-ABS((student* or undergrad* or sophom* or master*) W/3 (higher or college* or universit* or tertiar*))  #3 = #1 OR #2  #4 = TITLE-ABS(anxi* OR depress* OR psych* OR mental* OR stress* OR emotion* OR nervous* OR hypervigil* OR sleep* OR mood* OR "well-being" OR wellbeing OR wellness OR resilience OR coping OR cope OR competenc* OR "study achievement*" OR "self-esteem" OR "self-efficacy")  #5 = TITLE-ABS(prevent* OR promot* OR improv* OR intervent* OR favour* OR favor* OR counsel* OR councel* OR avoid* OR inhibit* OR program* OR support* OR skill*)  #6 = #3 AND #4 AND #5  #7 = TITLE-ABS ( "double-blind"  OR  "random* assigned"  OR  control )  #8 = #6 AND #7  #9 = TITLE ( review )  #10 = #6 AND #9  #11 = #8 OR #10 |
| --- |

The updated searches in Medline, PsycInfo, Eric and Scopus, December 1, 2015, yielded in total 352 records.
