# Supplementary material for: Effects of mental health interventions for students in higher education are sustainable over time: a systematic review and meta-analysis of randomized controlled trials
Source: PeerJ. 2018 Apr 2;6:e4598. doi: 10.7717/peerj.4598 (PMC5885977; doi:10.7717/peerj.4598)
Supplement: Supplemental Information 9 [file peerj-06-4598-s009.docx]

**Documentation of search strategies for grey literature**

Research question: Systematisk litteraturöversikt om psykisk hälsa hos universitetsstudenter.

Date: December 2015

Name of researcher: Regina Winzer, Folkhälsomyndigheten & Karolinska Institutet

Librarians: Anders Wändahl & Carl Gornitzki, University Library, Karolinska Institutet

Total number of hits: 1,203

Databases:

- Dart Europe 310
- OpenGrey 250
- Base Bielefeld 144
- Disserations & Theses 499

1. Dart Europe (theses)

Date: 2015-12-30

Number of hits: 310

| Nästan hopplös att söka i utan pregnanta ord. Ett försök nedan i alla fall. Gula länken går direkt till databasens träffar. Ibland sänker sökningen hela databasen.  (clinic* OR service* OR unit* OR counsel* OR center*) AND (student* OR undergrad* OR sophom* OR master*) AND (higher OR college* OR universit* OR tertiar*) AND (anxi* OR depress* OR psych* OR mental* OR stress* OR emotion* OR nervous* OR hypervigil* OR sleep* OR mood* OR well-being OR wellbeing OR Wellness OR resilience OR coping OR cope OR competenc* OR (study AND achievement*) OR self-esteem or self-efficacy) |
| --- |

2. OpenGrey (http://www.opengrey.eu/)

Date: 2015-12-29

Number of hits: 250

| abstract:((clinic* OR service* OR unit* OR counsel* OR center*)) AND abstract:(student* OR undergrad* OR sophom* OR master*) AND (higher OR college* OR universit* OR tertiar*) AND abstract:(anxi* OR depress* OR psych* OR mental* OR stress* OR emotion* OR nervous* OR hypervigil* OR sleep* OR mood* OR well-being OR wellbeing OR Wellness OR resilience OR coping OR cope OR competenc* OR (study AND achievement*) OR self-esteem or self-efficacy) |
| --- |

3. BASE Bielefeld http://www.base-search.net/

Date: 2015-12-30

Number of hits: 144

| N.A. Very difficult to use and describe the search strategy. |
| --- |

4. Dissertations & Theses (ProQuest)

Date: 2015-12-29

Number of hits: 499

| ((TI(student* OR campus OR universit* OR college*) AND TI(clinic* OR service* OR unit* OR counsel* OR center*)) OR (TI(student* OR undergrad* OR sophom* OR master*) AND TI(higher OR college* OR universit* OR tertiar*))) AND TI(anxi* OR depress* OR psych* OR mental* OR stress* OR emotion* OR nervous* OR hypervigil* OR sleep* OR mood* OR "well-being" OR wellbeing OR wellness OR resilience OR coping OR cope OR competenc* OR "study achievement*" OR "self-esteem" OR "self-efficacy") AND TI(prevent* OR promot* OR improv* OR intervent* OR favour* OR favor* OR counsel* OR councel* OR avoid* OR inhibit* OR program* OR support* OR skill*) |
| --- |
