# Supplementary material for: Effects of mental health interventions for students in higher education are sustainable over time: a systematic review and meta-analysis of randomized controlled trials
Source: PeerJ. 2018 Apr 2;6:e4598. doi: 10.7717/peerj.4598 (PMC5885977; doi:10.7717/peerj.4598)
Supplement: Supplemental Information 10 — 1. Coding for meta-analysis. 2. Original data recalculation. [file peerj-06-4598-s010.docx]

**Effects of mental health interventions for students in higher education are sustainable over time: A systematic review and meta-analysis of randomized controlled trials**

**CODING FOR META-ANALYSIS AND RECALCULATION OF ORIGINAL DATA**

**FOR STATA 13.1 (StataCorp, College Station, TX)**

**TABLE OF CONTENT:**

1. **Coding for meta-analysis** page 1
   1. Generating variables used in meta-analysis page 1
   2. Meta-coding for hierarchical outcomes
      (performed separately within data sets
      “Hierarchical negative outcomes” and
      “Hierarchical positive outcomes”) page 3
   3. Meta-coding for individual outcomes
      (performed in the dataset “Individual outcomes”) page 5
2. **Original data recalculation** pages 6

2.1 Comment, general formula and STATA coding for Hedge's g page 6

2.2. Hedges’ g recalculation based on follow-up means and SDs

reported in original studies page 7

2.3. Combined SMDs to avoid unit-of-analysis error page 22

2.3.1. Comment page 22

2.3.2. Coding for combining Hedges’g as explained in 2.3.1 page 22

2.4. Hierarchical outcomes page 24

2.4.1. STATA coding for hierarchical outcomes page 24

1. **CODING FOR META-ANALYSIS**
   1. **Generating variables used in meta-analysis**

*****Generate variable for follow-up lengths divided by: 3-6, 7-12, 13-18 month periods*****

gen FU_3cat=0 if lengt_followup_after_postint>=3 & lengt_followup_after_postint<=6

replace FU_3cat=1 if lengt_followup_after_postint>6 & lengt_followup_after_postint<=12

replace FU_3cat=2 if lengt_followup_after_postint>12 & lengt_followup_after_postint<=18

label define FU_3cat 0 " '3 - 6 MONTHS FOLLOW-UP POSTINTERVENTION' " 1 " '7 - 12 MONTHS FOLLOW-UP POSTINTERVENTION' " 2 " '13 - 18 MONTHS FOLLOW-UP POSTINTERVENTION' "

label values FU_3cat FU_3cat

label var FU_3cat "Categories for postint follow-up time: '3-6mo', '7-12mo', '13-18mo' "

*****Generate variable for percent of female participants numerical: 0 - females 40-60%; 1 - females >60%; 2 - females <40%; 3 - missing gender*****

gen gender_dichot=0 if percent_females_total<=60

replace gender_dichot=1 if percent_females_total>60

replace gender_dichot=2 if percent_females_total<=40

replace gender_dichot=3 if percent_females_total==.

label define gender_dichot 0 "females40-60%" 1 "females>60%" 2 "females<40%" 3 "missing gender"

label values gender_dichot gender_dichot

label var gender_dichot "Percent of females in total sample"

*****Generate variable for total sample size dichotomized: 0 - 100 participants or less; 1 - more than 100 participants *****

gen total_sample_size_dichot=0 if total_sample_size<=100

replace total_sample_size_dichot=1 if total_sample_size>100

label define total_sample_size_dichot 0 "size<=100" 1 "size>100"

label values total_sample_size_dichot total_sample_size_dichot

label var total_sample_size_dichot "Total sample size: 0=less or 100 pers, 1=more than 100"

*****Generate variable for countries dichotomized: 0 - US/Canada; 1 - Other countries *****

gen country_dichot=1

replace country_dichot=0 if region=="US/Canada"

label define country_dichot 0 "US/Canada" 1 "Other countries"

label values country_dichot country_dichot

label var country_dichot "Countries: 0= US/Canada, 1 = Other countries"

*****To account for different outcome groups (OUTCOME) and types of intervention (INTERVENTION)*****

foreach v of varlist OUTCOME {

egen `v'_INTERVENTION = group( `v' INTERVENTION) , label lname(name)

tab `v'_ INTERVENTION

decode `v'_ INTERVENTION , gen(`v'_ INTERVENTIONs)

}

*****To account for different types of intervention (INTERVENTION) outcome groups (OUTCOME)*****

foreach v of varlist INTERVENTION {

egen `v'_OUTCOME = group( `v' OUTCOME) , label lname(name)

tab `v'_OUTCOME

decode `v'_OUTCOME , gen(`v'_OUTCOMEs)

}

*****To account for different outcome groups (OUTCOME) and length of follow-up (FU_cat)*****

foreach v of varlist OUTCOME {

egen `v'_FU_3cat = group( `v' FU_3cat) , label lname(name)

tab `v'_FU_3cat

decode `v'_FU_3cat , gen(`v'_FU_3cats)

}

*****To account for different length of follow-up (FU_cat) and outcome groups (OUTCOME)*****

foreach v of varlist FU_3cat {

egen `v'_ OUTCOME = group( `v' OUTCOME) , label lname(name)

tab `v'_ OUTCOME

decode `v'_ OUTCOME , gen(`v'_ OUTCOMEs)

}

*****To account for different types of intervention (INTERVENTION) and length of follow-up (FU_cat)*****

foreach v of varlist INTERVENTION {

egen `v'_FU_3cat = group( `v' FU_3cat) , label lname(name)

tab `v'_FU_3cat

decode `v'_FU_3cat , gen(`v'_FU_3cats)

}

*****To account for different length of follow-up (FU_cat) and types of intervention (INTERVENTION)*****

foreach v of varlist FU_3cat {

egen `v'_ INTERVENTION = group( `v' INTERVENTION) , label lname(name)

tab `v'_ INTERVENTION

decode `v'_ INTERVENTION , gen(`v'_ INTERVENTIONs)

}

*****To account for different outcome_intervention groups (OUTCOME_INTERVENTION) and length of*** follow-up (FU_cat)**

foreach v of varlist OUT_INTERVENTION {

egen `v'_FU_3cat = group( `v' FU_3cat) , label lname(name)

tab `v'_FU_3cat

decode `v'_FU_3cat , gen(`v'_FU_3cats)

}

- 1. **META-CODING FOR HIERARCHICAL OUTCOMES (performed separately within data sets “Hierarchical negative outcomes” and “Hierarchical positive outcomes”)**

sort INTERVENTION pub_year idnr

******Main meta-analysis******

metan SMD LOWER_CI UPPER_CI, by (FU_3cat) randomi lcols( first_author_pubyr INTERVENTION) force xlabel(-1.5, -1.0, -0.5, 0, 0.5, 1.0, 1.5) textsize(100) nooverall

******Sub-group analyses******

***By type of intervention***

metan SMD LOWER_CI UPPER_CI if FU_3cat==0, by (INTERVENTION ) randomi lcols( first_author_pubyr INTERVENTION) force xlabel(-1.5, -1.0, -0.5, 0, 0.5, 1.0, 1.5) textsize(100) nooverall

***By intervention delivery level***

metan SMD LOWER_CI UPPER_CI if FU_3cat==0, by (delivery_level) randomi lcols( first_author_pubyr INTERVENTION) force xlabel(-1.5, -1.0, -0.5, 0, 0.5, 1.0, 1.5) textsize(100) nooverall

***By intervention format type***

metan SMD LOWER_CI UPPER_CI if FU_3cat==0, by (format_type ) randomi lcols( first_author_pubyr INTERVENTION) force xlabel(-1.5, -1.0, -0.5, 0, 0.5, 1.0, 1.5) textsize(100) nooverall

***By type of comparator group***

metan SMD LOWER_CI UPPER_CI if FU_3cat==0, by (control_type_group) randomi lcols( first_author_pubyr INTERVENTION) force xlabel(-1.5, -1.0, -0.5, 0, 0.5, 1.0, 1.5) textsize(100) nooverall

***By study quality***

metan SMD LOWER_CI UPPER_CI if FU_3cat==0, by (q_total_quality) randomi lcols( first_author_pubyr INTERVENTION) force xlabel(-1.5, -1.0, -0.5, 0, 0.5, 1.0, 1.5) textsize(100) nooverall

***By total study sample size***

metan SMD LOWER_CI UPPER_CI if FU_3cat==0, by (total_sample_size_dichot) randomi lcols( first_author_pubyr INTERVENTION) force xlabel(-1.5, -1.0, -0.5, 0, 0.5, 1.0, 1.5) textsize(100) nooverall

***By percentage of female participants***

metan SMD LOWER_CI UPPER_CI if FU_3cat==0, by (gender_dichot) randomi lcols( first_author_pubyr INTERVENTION) force xlabel(-1.5, -1.0, -0.5, 0, 0.5, 1.0, 1.5) textsize(100) nooverall

***By country where study was conducted***

metan SMD LOWER_CI UPPER_CI if FU_3cat==0, by (country_dichot) randomi lcols( first_author_pubyr INTERVENTION) force xlabel(-1.5, -1.0, -0.5, 0, 0.5, 1.0, 1.5) textsize(100) nooverall

******Influence analysis******

****for follow-up 3-6 months****

metaninf SMD LOWER_CI UPPER_CI if (FU_3cat==0) , randomi print label(namevar= first_author_pubyr )

****for follow-up 7-12 months****

metaninf SMD LOWER_CI UPPER_CI if (FU_3cat==1) , randomi print label(namevar= first_author_pubyr )

****for follow-up 13-18 months****

metaninf SMD LOWER_CI UPPER_CI if (FU_3cat==2) , randomi print label(namevar= first_author_pubyr )

*****Publication bias (performed separately for each follow-up period)*****

gen SESMD =( ( UPPER_CI ) - ( LOWER_CI )) / (2*invnorm(.975))

metafunnel SMD SESMD

metabias SMD SESMD, egger graph

metabias SMD SESMD, begg

- 1. **META-CODING FOR INDIVIDUAL OUTCOMES (performed in the dataset “Individual outcomes”)**

SMD LOWER_CI UPPER_CI if OUTCOME=="Depressive symptoms", by (FU_3cat) randomi lcols (first_author_pubyr INTER) force xlabel(-1.5, -1.0, -0.5, 0, 0.5, 1.0, 1.5) textsize(140) nooverall

SMD LOWER_CI UPPER_CI if OUTCOME=="Anxiety symptoms", by (FU_3cat) randomi lcols (first_author_pubyr INTER) force xlabel(-1.5, -1.0, -0.5, 0, 0.5, 1.0, 1.5) textsize(140) nooverall

SMD LOWER_CI UPPER_CI if OUTCOME=="Stress", by (FU_3cat) randomi lcols (first_author_pubyr INTER) force xlabel(-1.5, -1.0, -0.5, 0, 0.5, 1.0, 1.5) textsize(140) nooverall

SMD LOWER_CI UPPER_CI if OUTCOME=="Psychological distress", by (FU_3cat) randomi lcols (first_author_pubyr INTER) force xlabel(-1.5, -1.0, -0.5, 0, 0.5, 1.0, 1.5) textsize(140) nooverall

SMD LOWER_CI UPPER_CI if OUTCOME=="Self-reported worry", by (FU_3cat) randomi lcols (first_author_pubyr INTER) force xlabel(-1.5, -1.0, -0.5, 0, 0.5, 1.0, 1.5) textsize(140) nooverall

SMD LOWER_CI UPPER_CI if OUTCOME=="Quality of sleep", by (FU_3cat) randomi lcols (first_author_pubyr INTER) force xlabel(-1.5, -1.0, -0.5, 0, 0.5, 1.0, 1.5) textsize(140) nooverall

SMD LOWER_CI UPPER_CI if OUTCOME=="Passive coping", by (FU_3cat) randomi lcols (first_author_pubyr INTER) force xlabel(-1.5, -1.0, -0.5, 0, 0.5, 1.0, 1.5) textsize(140) nooverall

SMD LOWER_CI UPPER_CI if OUTCOME=="Academic performence ", by (FU_3cat) randomi lcols (first_author_pubyr INTER) force xlabel(-1.5, -1.0, -0.5, 0, 0.5, 1.0, 1.5) textsize(140) nooverall

SMD LOWER_CI UPPER_CI if OUTCOME=="Self-esteem ", by (FU_3cat) randomi lcols (first_author_pubyr INTER) force xlabel(-1.5, -1.0, -0.5, 0, 0.5, 1.0, 1.5) textsize(140) nooverall

SMD LOWER_CI UPPER_CI if OUTCOME=="Self-efficacy", by (FU_3cat) randomi lcols (first_author_pubyr INTER) force xlabel(-1.5, -1.0, -0.5, 0, 0.5, 1.0, 1.5) textsize(140) nooverall

SMD LOWER_CI UPPER_CI if OUTCOME=="Self-compassion ", by (FU_3cat) randomi lcols (first_author_pubyr INTER) force xlabel(-1.5, -1.0, -0.5, 0, 0.5, 1.0, 1.5) textsize(140) nooverall

SMD LOWER_CI UPPER_CI if (OUTCOME=="Mental well-being " | OUTCOME=="Subjective well-being "), by (FU_3cat) randomi lcols (first_author_pubyr INTER) force xlabel(-1.5, -1.0, -0.5, 0, 0.5, 1.0, 1.5) textsize(140) nooverall

SMD LOWER_CI UPPER_CI if OUTCOME=="Approach coping ", by (FU_3cat) randomi lcols (first_author_pubyr INTER) force xlabel(-1.5, -1.0, -0.5, 0, 0.5, 1.0, 1.5) textsize(140) nooverall

SMD LOWER_CI UPPER_CI if OUTCOME=="Happiness rating ", by (FU_3cat) randomi lcols (first_author_pubyr INTER) force xlabel(-1.5, -1.0, -0.5, 0, 0.5, 1.0, 1.5) textsize(140) nooverall

SMD LOWER_CI UPPER_CI if OUTCOME=="Stress management ", by (FU_3cat) randomi lcols (first_author_pubyr INTER) force xlabel(-1.5, -1.0, -0.5, 0, 0.5, 1.0, 1.5) textsize(140) nooverall

SMD LOWER_CI UPPER_CI if OUTCOME=="Resiliance ", by (FU_3cat) randomi lcols (first_author_pubyr INTER) force xlabel(-1.5, -1.0, -0.5, 0, 0.5, 1.0, 1.5) textsize(140) nooverall

**2. ORIGINAL DATA RECALCULATION**

**2.1. COMMENT:**

All recalculations below are based on the original mean and SDs reported in the studies for follow-up data. Because of the variety of instruments used for measuring outcomes, a standardized mean difference using Hedges’ g was chosen as a common effect size (ES) for conducting quantitative synthesis. ES was calculated separately at each post-intervention follow-up time point as a difference in means between intervention and control group, divided by the pooled within-group SD and incorporating a correction factor for small sample sizes (Borenstein et al. 2009).

**General formula and STATA coding for Hedge's g:**

**esizei #obs1 #mean1 #sd1 #obs2 #mean2 #sd2 [, options],**

where:

*esize* (STATA command) calculates effect sizes for comparing the difference between the means of a continuous variable for two groups

*#obs1* = number of participants in the intervention group

*#mean1* = mean reported at follow-up assessment for intervention group

*#sd1* = standard deviation for mean reported at follow-up assessment for intervention group

*#obs2* = number of participants in the control group

*#mean2* = mean reported at follow-up assessment for control group

*#sd2* = mean reported at follow-up assessment for control group

*all* (STATA option)= report all estimates of effect size, including **Hedges’ g**, Cohen’s d, Glass's Delta 1, Glass's Delta 2 and Point-Biserial’s r

Throughout the recalculations we kept the original direction of scales indicating the improvement of outcome measures. Thus, for negative outcomes ESs below zero pointed to superiority of the intervention group over the controls, while for positive outcomes, ESs above zero indicated that the results favored the intervention.

**2.2 Hedges’ g recalculation based on follow-up means and SDs reported in original studies**

(recalculated from the original studies)

***Chase JA, 2013***

**Intervention - Goalsetting+values (mind-body); Control - goalsetting only (active)**

**Outcome - Student performance (GPA), Follow-up post-intervention (6 months)**

**Raw data

esizei 51 3.16 0.56 48 3.23 0.66, all

** Intervention - Goalsetting+values (mind-body); Control - goalsetting only (active)**

** Outcome - Student performance (GPA), Follow-up post-intervention (6 months)**

**Adjusted data (for age, gender, Millenium Scholarship)*********

esizei 51 3.19 0.074 48 3.17 0.076, all

***Cheng M, 2015***

**Intervention - Meaning centered approach (psychoeduc); Control - no treatment (passive)**

** Outcome - Psychological well-being, Follow-up post-intervention (3 months)**

esizei 34 1.56 0.16 32 1.36 0.74, all

**Intervention - Meaning centered approach (psychoeduc); Control - no treatment (passive)**

** Outcome - Depression, Follow-up post-intervention (3 months)**

esizei 34 1.64 0.19 32 1.48 0.40, all

**Intervention - Meaning centered approach (psychoeduc); Control - no treatment (passive)**

**Outcome - Anxiety, Follow-up post-intervention (3 months)**

esizei 34 1.45 0.38 32 1.38 0.19, all

** Intervention - Meaning centered approach (psychoeduc); Control - no treatment (passive)**

** Outcome - Self-esteem (SES), Follow-up post-intervention (3 months)**

esizei 34 3.14 0.31 32 3.10 0.42, all

***Erogul M, 2014***

** Intervention - Mindfulness based stress reduction (mind-body); Control - no treatment (passive)**

** Outcome - Perceived stress (PSS), Follow-up post-intervention (6 months)**

esizei 28 14.9 6.6 29 18.4 6.9, all

**Intervention - Mindfulness based stress reduction (mind-body); Control - no treatment (passive)**

** Outcome - Self-compassion (SCS), Follow-up post-intervention (6 months)**

esizei 28 3.7 0.53 29 3.1 0.6, all

** Intervention - Mindfulness based stress reduction (mind-body); Control - no treatment (passive)**

** Outcome - Resilience (RS-14), Follow-up post-intervention (6 months)**

esizei 28 82.4 9.8 29 77.3 12.5, all

***Fontana A, 1999***

**Intervention - Peer-led stress inoculation training (CBT); Control - WL (passive)**

**Outcome - State anxiety (STAI), Follow-up post-intervention (6 months)**

esizei 15 32.87 7.54 12 36.27 14.20, all

***Franklin J, 2012***

** Intervention - PAAL enhanced coaching (psychoedu); Control - Self-regulation (active)**

** Outcome - Academic performance (GPA), Follow-up post-intervention (12 months)**

esizei 27 71.04 10.52 25 62.98 10.99, all

** Intervention - Self-regulation (psychoedu); Control - no treatment (passive)**

** Outcome - Academic performance (GPA), Follow-up post-intervention (12 months)**

esizei 25 62.98 10.99 2183 61.29 16.34 , all

** Intervention - PAAL enhanced coaching (psychoedu); Control - no treatment (passive)**

** Outcome - Academic performance (GPA), Follow-up post-intervention (12 months)**

esizei 27 71.04 10.52 2183 61.29 16.34 , all

** Intervention - PAAL enhanced coaching (psychoedu); Control - Self-regulation (active)**

** Outcome - Academic performance (GPA), Follow-up post-intervention (18 months)**

esizei 25 70.97 10.26 24 62.11 10.14, all

**Intervention - Self-regulation (psychoedu); Control - no treatment (passive)**

**Outcome - Academic performance (GPA), Follow-up post-intervention (18 months)**

esizei 24 62.11 10.14 2063 60.48 16.07 , all

**Intervention - PAAL enhanced coaching (psychoedu); Control - no treatment (passive)**

** Outcome - Academic performance (GPA), Follow-up post-intervention (18 months)**

esizei 25 70.97 10.26 2063 60.48 16.07 , all

***Gortner E-M, 2006***

**Intervention - Expressive writing low suppression (mind-bodt); Control - Control writing low suppression (active)**

**Outcome - Depressive symptoms (BDI), Follow-up post-intervention (6 months)**

esizei 28 7.00 6.30 14 4.21 4.71, all

**Intervention - Expressive writing high suppression (mind-bodt); Control - Control writing high suppression (active)**

**Outcome - Depressive symptoms (BDI), Follow-up post-intervention (6 months)**

esizei 24 4.46 2.98 24 6.25 4.78, all

***Hamdan-Mansour A, 2009***

**Intervention - Cognitive behavioral intervention (CBT); Control - No treatment (passive)**

**Outcome - Perceived stress (PSS), Follow-up post-intervention (3 months)**

esizei 44 15.5 6.7 40 20.5 6.1, all

**Intervention - Cognitive behavioral intervention (CBT); Control - No treatment (passive)**

**Outcome - Depression (BDI), Follow-up post-intervention (3 months)**

esizei 44 12.7 9.4 40 17.8 9.9, all

**Intervention - Cognitive behavioral intervention (CBT); Control - No treatment (passive)**

**Outcome - Approach coping (WCQ approach coping strategies composite), Follow-up post-intervention (3 months)**

esizei 44 60.7 11.8 40 48.8 13.7, all

**Intervention - Cognitive behavioral intervention (CBT); Control - No treatment (passive)**

**Outcome - Avoidance coping (WCQ avoidance coping strategies composite), Follow-up post-intervention (3 months)**

esizei 44 30.4 5.5 40 35.6 4.8, all

***Higgins D, 2006***

**Intervention - Cognitive behavioral intervention (CBT); Control - No treatment (passive)**

**Outcome - Self-reported worry (PSWQ), Follow-up post-intervention (6 months)**

**Data from table 5 thesis, page 85. Follow-up 6 months postintervention**

esizei 25 49.72 7.59 32 47.50 10.42, all

**Intervention - Cognitive behavioral intervention (CBT); Control - No treatment (passive)**

**Outcome - Self-reported worry (PSWQ), Follow-up post-intervention (12 months)**

**Data from table 6 thesis, page 88. Follow-up 12 months postintervention**

esizei 21 51.00 8.49 25 48.12 11.76, all

**Intervention - Cognitive behavioral intervention (CBT); Control - No treatment (passive)**

**Outcome - Generalized anxiety disorder (GADQ), Follow-up post-intervention (6 months)**

**Data from table 5 thesis, page 85. Follow-up 6 months postintervention**

esizei 25 11.86 5.95 32 11.06 6.98, all

**Intervention - Cognitive behavioral intervention (CBT); Control - No treatment (passive)**

**Outcome - Generalized anxiety disorder (GADQ), Follow-up post-intervention (12 months)**

**Data from table 6 thesis, page 88. Follow-up 12 months postintervention**

esizei 21 13.48 6.30 25 11.56 6.66, all

**Intervention - Cognitive behavioral intervention (CBT); Control - No treatment (passive)**

**Outcome - Depressive symptoms (BDI), Follow-up post-intervention (6 months)**

**Data from table 5 thesis, page 85. Follow-up 6 months postintervention**

esizei 25 10.04 9.70 32 11.66 9.75, all

**Intervention - Cognitive behavioral intervention (CBT); Control - No treatment (passive)**

**Outcome - Depressive symptoms (BDI), Follow-up post-intervention (12 months)**

**Data from table 6 thesis, page 88. Follow-up 12 months postintervention**

esizei 21 10.38 8.86 25 11.76 9.92, all

**Intervention - Cognitive behavioral intervention (CBT); Control - No treatment (passive)**

**Outcome - Anxiety symptoms (BAI), Follow-up post-intervention (6 months)**

**Data from table 5 thesis, page 85. Follow-up 6 months postintervention.**

esizei 25 7.84 5.62 32 7.56 6.84, all

**Intervention - Cognitive behavioral intervention (CBT); Control - No treatment (passive)**

**Outcome - Anxiety symptoms (BAI), Follow-up post-intervention (12 months)**

**Data from table 6 thesis, page 88. Follow-up 12 months postntervention**

esizei 21 8.57 5.24 25 8.76 6.82, all

***Jones M, 2000***

**Intervention - Multi-modal stress reduction and stress management (CBT); Control - WL (passive)**

**Outcome - General mental health (GHQ-30), Follow-up post-intervention (3 months)**

**Data from table 6.9 **

esizei 39 17.85 10.53 33 30.15 14.13 , all

**Intervention - Multi-modal stress reduction and stress management (CBT); Control - WL (passive)**

**Outcome - State anxiety (STAI), Follow-up post-intervention (3 months)**

**Data from table 6.9 **

esizei 39 31.62 8.48 34 39.44 11.34 , all

**OBS! Intervention - Multi-modal stress reduction and stress management (CBT); Control - WL (passive)**

**Outcome - Trait anxiety (STAI), Follow-up post-intervention (3 months)**

**Data from table 6.9 **

esizei 39 36.45 9.03 34 42.45 9.62 , all

**Intervention - Multi-modal stress reduction and stress management (CBT); Control - WL (passive)**

**Outcome - Depressive symptoms (BDI), Follow-up post-intervention (3 months)**

**Data from table 6.9 **

esizei 39 3.85 4.88 34 8.56 5.87, all

**Intervention - Multi-modal stress reduction and stress management (CBT); Control - WL (passive)**

**Outcome - General coping (The "Ways of coping questionnaire"), Follow-up post-intervention (3 months)**

**Data from table 6.9 **

esizei 39 16.23 7.83 34 17.91 7.78, all

**Intervention - Multi-modal stress reduction and stress management (CBT); Control - WL (passive)**

**Outcome - Direct coping (The "Ways of coping questionnaire"), Follow-up post-intervention (3 months)**

**Data from table 6.9 **

esizei 39 1.82 3.92 34 -1.32 4.03, all

**Intervention - Multi-modal stress reduction and stress management (CBT); Control - WL (passive)**

**Outcome - Supression (The "Ways of coping questionnaire"), Follow-up post-intervention (3 months)**

**Data from table 6.9 **

esizei 39 0.08 1.82 34 -0.24 1.79, all

**Intervention - Multi-modal stress reduction and stress management (CBT); Control - WL (passive)**

**Outcome - Stress (BSSI), Follow-up post-intervention (3 months)**

**Data from table 6.9 **

esizei 39 55.77 13.91 34 67.23 16.07, all

***Kanji N, 2006***

**Intervention - Autogenic training (mind-body); Control - attention control (active)**

**Outcome - State anxiety (STAI), Follow-up post-intervention (3 months)**

**ITT analysis**

esizei 32 38.1 13.5 30 43.4 15.8, all

**Intervention - Autogenic training (mind-body); Control - attention control (active)**

**Outcome - State anxiety (STAI), Follow-up post-intervention (6 months)**

**ITT analysis**

esizei 32 33.7 10.1 30 42.8 15.3, all

**Intervention - Autogenic training (mind-body); Control - attention control (active)**

**Outcome - State anxiety (STAI), Follow-up post-intervention (9 months)**

**ITT analysis**

esizei 32 30.1 8.1 30 37.3 9.5, all

**Intervention - Autogenic training (mind-body); Control - attention control (active)**

**Outcome - State anxiety (STAI), Follow-up post-intervention (12 months)**

**ITT analysis**

esizei 32 35.1 11.2 30 40.0 8.9, all

**Intervention - Autogenic training (mind-body); Control - time control (passive)**

**Outcome - State anxiety (STAI), Follow-up post-intervention (3 months)**

**ITT analysis**

esizei 32 38.1 13.5 31 43.5 11.0, all

**Intervention - Autogenic training (mind-body); Control - time control (passive)**

**Outcome - State anxiety (STAI), Follow-up post-intervention (6 months)**

**ITT analysis**

esizei 32 33.7 10.1 31 39.1 12.7, all

**Intervention - Autogenic training (mind-body); Control - time control (passive)**

**Outcome - State anxiety (STAI), Follow-up post-intervention (9 months)**

**ITT analysis**

esizei 32 30.1 8.1 31 39.6 9.5, all

**Intervention - Autogenic training (mind-body); Control - time control (passive)**

**Outcome - State anxiety (STAI), Follow-up post-intervention (12 months)**

**ITT analysis**

esizei 32 35.1 11.2 31 38.1 8.9, all

**Intervention - Autogenic training (mind-body); Control - attention control (active)**

**Outcome - Trait anxiety (STAI), Follow-up post-intervention (3 months)**

**ITT analysis**

esizei 32 36.9 10.1 30 44.8 10.7, all

**Intervention - Autogenic training (mind-body); Control - attention control (active)**

**Outcome - Trait anxiety (STAI), Follow-up post-intervention (6 months)**

**ITT analysis**

esizei 32 34.2 7.9 30 42.7 10.4, all

**Intervention - Autogenic training (mind-body); Control - attention control (active)**

**Outcome - Trait anxiety (STAI), Follow-up post-intervention (9 months)**

**ITT analysis**

esizei 32 33.2 5.9 30 38.9 5.2, all

**Intervention - Autogenic training (mind-body); Control - attention control (active)**

**Outcome - Trait anxiety (STAI), Follow-up post-intervention (12 months)**

**ITT analysis**

esizei 32 35.4 9.4 30 41.3 7.9 , all

**Intervention - Autogenic training (mind-body); Control - time control (passive)**

**Outcome - Trait anxiety (STAI), Follow-up post-intervention (3 months)**

**ITT analysis**

esizei 32 36.9 10.1 31 42.7 6.3, all

**Intervention - Autogenic training (mind-body); Control - time control (passive)**

**Outcome - Trait anxiety (STAI), Follow-up post-intervention (6 months)**

**ITT analysis**

esizei 32 34.2 7.9 31 39.7 6.9, all

**Intervention - Autogenic training (mind-body); Control - time control (passive)**

**Outcome - Trait anxiety (STAI), Follow-up post-intervention (9 months)**

**ITT analysis**

esizei 32 33.2 5.9 31 40.3 8.2, all

**Intervention - Autogenic training (mind-body); Control - time control (passive)**

**Outcome - Trait anxiety (STAI), Follow-up post-intervention (12 months)**

**ITT analysis**

esizei 32 35.4 9.4 31 39.0 7.5 , all

***Kattelmann K, 2011***

**Intervention - Young Adults Eating and Active for Health (psychedu); Control - WL (passive)**

**Outcome - Perceived stress (PSS-14), Follow-up post-intervention (12 months)**

**All analyses are made on FU completers

esizei 497 22.9 7.6 476 23.5 8.1 , all

***Kenardy J, 2006***

**Intervention - Online Anxiety Prevention Program (CBT); Control - WL (passive)**

**Outcome - DEpressive symptoms (CES-D), Follow-up post-intervention (4,5 months)**

**All analyses are made on FU completers **

esizei 19 15.58 5.03 23 20.78 7.93 , all

**Intervention - Online Anxiety Prevention Program (CBT); Control - WL (passive)**

**Outcome - Anxiety (ASI), Follow-up post-intervention (4,5 months)**

**All analyses are made on FU completers**

esizei 19 17.63 7.14 23 21.39 12.89 , all

***Li M, 2015***

**Intervention - Baduanjin exercise (mind-body); Control - exercise as usual (passive)**

**Outcome - Self-efficacy (GSES), Follow-up post-intervention (3 months)**

**ITT analysis**

esizei 101 2.50 0.45 105 2.61 0.46 , all

**Intervention - Baduanjin exercise (mind-body); Control - exercise as usual (passive)**

**Outcome - Stress (CPSS), Follow-up post-intervention (3 months)**

**ITT analysis**

esizei 101 22.72 5.75 105 23.22 5.72 , all

**Intervention - Baduanjin exercise (mind-body); Control - exercise as usual (passive)**

**Outcome - Self-esteem (SES), Follow-up post-intervention (3 months)**

**ITT analysis**

esizei 101 30.81 3.45 105 31.00 3.71 , all

**Intervention - Baduanjin exercise (mind-body); Control - exercise as usual (passive)**

**Outcome - Quality of sleep (PSQI), Follow-up post-intervention (3 months)**

**ITT analysis**

esizei 101 3.56 1.62 105 3.79 1.80 , all

***Mak W, 2015***

**Intervention - HAPA-Mindfulness (mind-body); Control - exercise as usual (passive)**

**Outcome - Mental well-being (WHO-5), Follow-up post-intervention (3 months)**

**ITT analysis**

esizei 105 14.51 5.05 79 16.22 5.74, all

**Intervention - HAPA-Mindfulness (mind-body); Control - exercise as usual (passive)**

**Outcome - Perceived stress (PSS-10), Follow-up post-intervention (3 months)**

**ITT analysis**

esizei 105 1.67 0.62 79 1.63 0.70, all

**Intervention - HAPA-Mindfulness (mind-body); Control - exercise as usual (passive)**

**Outcome - Depression (DASS-21), Follow-up post-intervention (3 months)**

**ITT analysis**

esizei 105 6.88 7.70 79 5.90 8.12, all

**Intervention - HAPA-Mindfulness (mind-body); Control - exercise as usual (passive)**

**Outcome - Anxiety (DASS-21), Follow-up post-intervention (3 months)**

**ITT analysis**

esizei 105 6.46 6.60 79 5.72 7.64, all

**Intervention - HAPA-Mindfulness (mind-body); Control - exercise as usual (passive)**

**Outcome - Stress (DASS-21), Follow-up post-intervention (3 months)**

**ITT analysis**

esizei 105 10.72 9.01 79 9.19 9.35, all

**Intervention - Basic Mindfulness (mind-body); Control - exercise as usual (passive)**

**Outcome - Mental well-being (WHO-5), Follow-up post-intervention (3 months)**

**ITT analysis**

esizei 104 14.47 5.21 79 16.22 5.74, all

**Intervention - Basic Mindfulness (mind-body); Control - exercise as usual (passive)**

**Outcome - Perceived stress (PSS-10), Follow-up post-intervention (3 months)**

**ITT analysis**

esizei 104 1.70 0.53 79 1.63 0.70, all

**Intervention - Basic Mindfulness (mind-body); Control - exercise as usual (passive)**

**Outcome - Depression (DASS-21), Follow-up post-intervention (3 months)**

**ITT analysis**

esizei 104 6.81 7.16 79 5.90 8.12, all

**Intervention - Basic Mindfulness (mind-body); Control - exercise as usual (passive)**

**Outcome - Anxiety (DASS-21), Follow-up post-intervention (3 months)**

**ITT analysis**

esizei 104 7.29 6.36 79 5.72 7.64, all

**Intervention - Basic Mindfulness (mind-body); Control - exercise as usual (passive)**

**Outcome - Stress (DASS-21), Follow-up post-intervention (3 months)**

**ITT analysis**

esizei 104 11.50 8.93 79 9.19 9.35, all

***Pachankis J, 2010***

**Intervention - Combined expressive writing and writing+reading (mind-body); Control - neutral writing (active)**

**Outcome - Psychopathology symptoms (SCL-90), Follow-up post-intervention (3 months)**

**Data from table (all participants are FU completers)**

esizei 52 151.78 5.82 25 155.33 8.42, all

**Intervention - Combined expressive writing and writing+reading (mind-body); Control - neutral writing (active)**

**Outcome - Depressive symptoms (CES-D), Follow-up post-intervention (3 months)**

**Data from table (all participants are FU completers)**

esizei 52 35.35 1.26 25 34.90 1.84, all

**Intervention - Combined expressive writing and writing+reading (mind-body); Control - neutral writing (active)**

**Outcome - Self-esteem (RSES), Follow-up post-intervention (3 months)**

**Data from table (all participants are FU completers)**

esizei 52 19.11 0.55 25 18.38 0.81, all

***Peden A, 2001***

**Intervention - Cognitive-behavioral intervention (CBT); Control - no treatment (passive)**

**Outcome - Depressive symptoms (CES-D), Follow-up post-intervention (6 months)**

**Data from table **

esizei 35 10.48 6.32 31 19.64 11.13, all

**Intervention - Cognitive-behavioral intervention (CBT); Control - no treatment (passive)**

**Outcome - Depressive symptoms (CES-D), Follow-up post-intervention (18 months)**

**Data from table **

esizei 22 9.86 6.10 21 12.76 9.42, all

**Intervention - Cognitive-behavioral intervention (CBT); Control - no treatment (passive)**

**Outcome - Depressive symptoms (BDI), Follow-up post-intervention (6 months)**

**Data from table **

esizei 35 5.62 4.87 31 14.38 9.70, all

**Intervention - Cognitive-behavioral intervention (CBT); Control - no treatment (passive)**

**Outcome - Depressive symptoms (BDI), Follow-up post-intervention (18 months)**

**Data from table **

esizei 23 5.91 4.85 21 8.85 8.30, all

**Intervention - Cognitive-behavioral intervention (CBT); Control - no treatment (passive)**

**Outcome - Self-esteem (RSE), Follow-up post-intervention (6 months)**

**Data from table **

esizei 35 32.28 6.35 31 24.64 7.61, all

**Intervention - Cognitive-behavioral intervention (CBT); Control - no treatment (passive)**

**Outcome - Self-esteem (RSE), Follow-up post-intervention (18 months)**

**Data from table **

esizei 23 31.08 8.08 21 28.71 7.72, all

***Reavley N, 2014***

**Intervention - MindWise (psychoedu); Control - no treatment (passive)**

**Outcome - Psychological stress (K6), Follow-up post-intervention (6 months)**

**NON-IMPUTED RESULTS from table**

esizei 284 11.70 4.69 209 11.00 3.62, all

**Intervention - MindWise (psychoedu); Control - no treatment (passive)**

**Outcome - Psychological stress (K6), Follow-up post-intervention (18 months)**

**NON-IMPUTED RESULTS from table!**

esizei 212 10.62 3.89 137 10.66 3.88, all

**Intervention - MindWise (psychoedu); Control - no treatment (passive)**

**Outcome - Psychological stress (K6), Follow-up post-intervention (6 months)**

**IMPUTED RESULTS from table!**

esizei 426 11.67 5.08 341 11.20 4.85, all

**Intervention - MindWise (psychoedu); Control - no treatment (passive)**

**Outcome - Psychological stress (K6), Follow-up post-intervention (18 months)**

**IMPUTED RESULTS from table!**

esizei 426 10.53 5.32 341 10.67 5.21, all

***Rohde P, 2014***

**Intervention - Cognitive behaviour intervention (CGT); Control - Brochure control (passive)**

**Outcome - Depressive symptoms (K-SADS), Follow-up post-intervention (6 months)**

**ITT RESULTS**

esizei 27 1.26 0.33 33 1.27 0.37, all

**Intervention - Cognitive behaviour intervention (CGT); Control - Brochure control (passive)**

**Outcome - Depressive symptoms (K-SADS), Follow-up post-intervention (12 months)**

**ITT RESULTS**

esizei 27 1.27 0.34 33 1.18 0.30, all

**Intervention - Cognitive behaviour intervention (CGT); Control - Bibliotherapy (active)**

**Outcome - Depressive symptoms (K-SADS), Follow-up post-intervention (6 months)**

**ITT RESULTS**

esizei 27 1.26 0.33 22 1.30 0.36, all

**Intervention - Cognitive behaviour intervention (CGT); Control - Bibliotherapy (active)**

**Outcome - Depressive symptoms (K-SADS), Follow-up post-intervention (12 months)**

**ITT RESULTS**

esizei 27 1.27 0.34 22 1.16 0.35, all

***Seligman M, 1999***

**Intervention - Prevention workshop (CBT); Control - no treatment (passive)**

**Outcome - Depressive symptoms (HDRS), Follow-up post-intervention (6 months)**

**Data from table**

esizei 106 2.2 2.6 117 2.3 2.4, all

**Intervention - Prevention workshop (CBT); Control - no treatment (passive)**

**Outcome - Depressive symptoms (BDI), Follow-up post-intervention (6 months)**

** Data from table**

esizei 106 2.9 3.9 117 3.3 3.3, all

**Intervention - Prevention workshop (CBT); Control - no treatment (passive)**

**Outcome - Anxiety symptoms (HARS), Follow-up post-intervention (6 months)**

**Data from table**

esizei 106 2.4 2.8 115 2.6 2.7, all

**ntervention - Prevention workshop (CBT); Control - no treatment (passive)**

**Outcome - Anxiety symptoms (BAI), Follow-up post-intervention (6 months)**

**Data from table**

esizei 106 2.6 3.6 117 2.8 3.1, all

**Intervention - Prevention workshop (CBT); Control - no treatment (passive)**

**Outcome - Depressive symptoms (HDRS), Follow-up post-intervention (12 months)**

**Data from table**

esizei 105 2.1 2.4 117 2.3 2.5, all

**Intervention - Prevention workshop (CBT); Control - no treatment (passive)**

**Outcome - Depressive symptoms (BDI), Follow-up post-intervention (12 months)**

**Data from table**

esizei 103 2.2 3.3 116 3.1 3.8, all

**Intervention - Prevention workshop (CBT); Control - no treatment (passive)**

**Outcome - Anxiety symptoms (HARS), Follow-up post-intervention (12 months)**

**Data from table**

esizei 105 2.6 2.5 117 3.0 2.8, all

**Intervention - Prevention workshop (CBT); Control - no treatment (passive)**

**Outcome - Anxiety symptoms (BAI), Follow-up post-intervention (12 months)**

**Data from table**

esizei 103 2.5 2.8 116 3.2 3.8, all

**Intervention - Prevention workshop (CBT); Control - no treatment (passive)**

**Outcome - Depressive symptoms (HDRS), Follow-up post-intervention (18 months)**

**Data from table**

esizei 101 2.0 2.2 114 2.4 3.3, all

**Intervention - Prevention workshop (CBT); Control - no treatment (passive)**

**Outcome - Depressive symptoms (BDI), Follow-up post-intervention (18 months)**

**Data from table**

esizei 97 2.0 2.8 110 3.4 3.9, all

**Intervention - Prevention workshop (CBT); Control - no treatment (passive)**

**Outcome - Anxiety symptoms (HARS), Follow-up post-intervention (18 months)**

**Data from table**

esizei 101 2.2 2.5 114 2.8 3.6, all

**Intervention - Prevention workshop (CBT); Control - no treatment (passive)**

**Outcome - Anxiety symptoms (BAI), Follow-up post-intervention (18 months)**

**Data from table**

esizei 97 1.5 2.2 110 3.0 3.9, all

**Intervention - Prevention workshop (CBT); Control - no treatment (passive)**

**Outcome - Depressive symptoms (HDRS), Follow-up post-intervention (24 months)**

**Data from table**

esizei 96 2.6 2.9 107 2.2 2.7, all

**Intervention - Prevention workshop (CBT); Control - no treatment (passive)**

**Outcome - Depressive symptoms (BDI), Follow-up post-intervention (24 months)**

**Data from table**

esizei 84 2.0 2.8 110 3.4 3.9, all

**Intervention - Prevention workshop (CBT); Control - no treatment (passive)**

**Outcome - Anxiety symptoms (HARS), Follow-up post-intervention (24 months)**

**Data from table**

esizei 96 2.5 2.9 108 2.4 2.7, all

**Intervention - Prevention workshop (CBT); Control - no treatment (passive)**

**Outcome - Anxiety symptoms (BAI), Follow-up post-intervention (24 months)**

**Data from table**

esizei 84 2.6 2.9 99 2.7 4.3, all

**Intervention - Prevention workshop (CBT); Control - no treatment (passive)**

**Outcome - Depressive symptoms (HDRS), Follow-up post-intervention (30 months)**

**Data from table**

esizei 96 2.6 3.8 109 2.2 2.4, all

**Intervention - Prevention workshop (CBT); Control - no treatment (passive)**

**Outcome - Depressive symptoms (BDI), Follow-up post-intervention (30 months)**

**Data from table**

esizei 85 2.4 4.2 102 2.8 3.5, all

**Intervention - Prevention workshop (CBT); Control - no treatment (passive)**

**Outcome - Anxiety symptoms (HARS), Follow-up post-intervention (30 months)**

**Data from table**

esizei 96 2.9 3.6 109 2.7 3.1, all

**Intervention - Prevention workshop (CBT); Control - no treatment (passive)**

**Outcome - Anxiety symptoms (BAI), Follow-up post-intervention (30 months)**

**Data from table**

esizei 85 2.0 2.8 102 2.5 3.0, all

**Intervention - Prevention workshop (CBT); Control - no treatment (passive)**

**Outcome - Depressive symptoms (HDRS), Follow-up post-intervention (36 months)**

**Data from table**

esizei 99 2.1 2.4 109 2.4 2.7, all

**Intervention - Prevention workshop (CBT); Control - no treatment (passive)**

**Outcome - Depressive symptoms (BDI), Follow-up post-intervention (36 months)**

**Data from table**

esizei 100 1.7 2.5 110 2.6 3.6, all

**Intervention - Prevention workshop (CBT); Control - no treatment (passive)**

**Outcome - Anxiety symptoms (HARS), Follow-up post-intervention (36 months)**

**Data from table**

esizei 100 2.4 2.9 109 2.5 2.6, all

**Intervention - Prevention workshop (CBT); Control - no treatment (passive)**

**Outcome - Anxiety symptoms (BAI), Follow-up post-intervention (36 months)**

**Data from table**

esizei 100 1.7 2.7 110 2.3 2.9, all

***Seligman M, 2007***

**Intervention - Cognitive-behaviour workshop (CBT); Control - no treatment (passive)**

**Outcome - Depressive symptoms (BDI), Follow-up post-intervention (4 months)**

**Data from table**

esizei 98 7.6 4.7 123 10.8 5.4, all

**Intervention - Cognitive-behaviour workshop (CBT); Control - no treatment (passive)**

**Outcome - Depressive symptoms (BDI), Follow-up post-intervention (6 months)**

**Data from table**

esizei 92 8.1 6.2 120 9.5 6.0, all

**Intervention - Cognitive-behaviour workshop (CBT); Control - no treatment (passive)**

**Outcome - Anxiety symptoms (BAI), Follow-up post-intervention (4 months)**

**Data from table**

esizei 98 6.9 4.8 123 9.4 5.8, all

**Intervention - Cognitive-behaviour workshop (CBT); Control - no treatment (passive)**

**Outcome - Anxiety symptoms (BAI), Follow-up post-intervention (6 months)**

**Data from table**

esizei 92 6.3 5.8 120 7.0 5.4, all

**Intervention - Cognitive-behaviour workshop (CBT); Control - no treatment (passive)**

**Outcome - Average happiness rating (Fordyce Emotions Questionnaire - rating), Follow-up post-intervention (4 months)**

**Data from table**

esizei 98 6.7 1.3 121 6.3 1.6 , all

**Intervention - Cognitive-behaviour workshop (CBT); Control - no treatment (passive)**

**Outcome - Time feel happy% (Fordyce Emotions Questionnaire - % of time), Follow-up post-intervention (4 months)**

**Data from table**

esizei 98 45.8 20.0 121 46.0 15.3 , all

***Shapiro S, 2011***

**Intervention - MBSR (mind-body); Control - WL (passive)**

**Outcome - Perceived stress (PSS), Follow-up post-intervention (12 months)**

**Data from table**

esizei 15 1.59 0.87 15 1.77 0.45, all

**Intervention - MBSR (mind-body); Control - WL (passive)**

**Outcome - Subjective well-being (Overall Subjective Well-being score), Follow-up post-intervention (12 months)**

**Data from table**

esizei 15 3.78 1.25 15 3.29 1.27, all

**Intervention - MBSR (mind-body); Control - WL (passive)**

**Outcome - Self-compassion (SCS), Follow-up post-intervention (12 months)**

**Data from table**

esizei 15 0.78 0.17 15 0.72 0.16, all

***Vázquez F, 2012***

**Intervention - CBT (CBT); Control - Relaxation training (active)**

**Outcome - Depressive symptoms (CES-D), Follow-up post-intervention (3 months)**

**ITT analysis!**

esizei 70 16.62 11.87 63 16.84 11.52, all

**Intervention - CBT (CBT); Control - Relaxation training (active)**

**Outcome - Depressive symptoms (CES-D), Follow-up post-intervention (6 months)**

**ITT analysis!**

esizei 70 17.74 11.85 63 16.44 12.49, all

**Intervention - CBT (CBT); Control - Relaxation training (active)**

**Outcome - Anxietysymptoms (BAI), Follow-up post-intervention (3 months)**

**ITT analysis!**

esizei 70 12.65 9.29 63 9.95 7.24, all

**Intervention - CBT (CBT); Control - Relaxation training (active)**

**Outcome - Anxietysymptoms (BAI), Follow-up post-intervention (6 months)**

**ITT analysis!**

esizei 70 10.89 8.75 63 11.59 9.34, all

***Yang W, 2015***

**Intervention - Attention bias modification (CBT); Control - Placebo control (passive)**

**Outcome - Depressive symptoms (BDI-II), Follow-up post-intervention (3 months)**

**ITT analysis!**

esizei 27 9.56 4.83 27 14.41 7.12, all

**Intervention - Attention bias modification (CBT); Control - Placebo control (passive)**

**Outcome - Depressive symptoms (BDI-II), Follow-up post-intervention (7 months)**

**ITT analysis!**

esizei 27 8.95 4.68 27 13.20 7.95, all

**Intervention - Attention bias modification (CBT); Control - assessment only (passive)**

**Outcome - Depressive symptoms (BDI-II), Follow-up post-intervention (3 months)**

**ITT analysis!**

esizei 27 9.56 4.83 23 13.38 5.97, all

**Intervention - Attention bias modification (CBT); Control - assessment only (passive)**

**Outcome - Depressive symptoms (BDI-II), Follow-up post-intervention (7 months)**

**ITT analysis!**

esizei 27 8.95 4.68 23 10.56 7.35, all

**Intervention - Attention bias modification (CBT); Control - Placebo control (passive)**

**Outcome - Anxiety symptoms (STAI-I, Follow-up post-intervention (3 months)**

**ITT analysis!**

esizei 27 44.74 5.20 27 47.41 7.42, all

**Intervention - Attention bias modification (CBT); Control - Placebo control (passive)**

**Outcome - Anxiety symptoms (STAI-I, Follow-up post-intervention (7 months)**

**ITT analysis!**

esizei 27 47.87 5.31 27 48.25 6.38, all

**Intervention - Attention bias modification (CBT); Control - assessment only (passive)**

**Outcome - Anxiety symptoms (STAI-I, Follow-up post-intervention (3 months)**

**ITT analysis!**

esizei 27 44.74 5.20 23 46.82 7.86, all

**Intervention - Attention bias modification (CBT); Control - assessment only (passive)**

**Outcome - Anxiety symptoms (STAI-I, Follow-up post-intervention (7 months)**

**ITT analysis!**

esizei 27 47.87 5.31 23 45.94 6.79, all

***Zheng G, 2015***

**Intervention - Tai Chi Chuan (mind-body); Control - excersise as usual (passive)**

**Outcome - Self-efficacy (GSES), Follow-up post-intervention (3 months)**

**ITT analysis!**

esizei 95 2.59 0.48 103 2.55 0.47, all

**Intervention - Tai Chi Chuan (mind-body); Control - excersise as usual (passive)**

**Outcome - Stress (CPSS), Follow-up post-intervention (3 months)**

**ITT analysis!**

esizei 95 22.55 6.44 103 23.47 6.65, all

**Intervention - Tai Chi Chuan (mind-body); Control - excersise as usual (passive)**

**Outcome - Self-esteem (SES), Follow-up post-intervention (3 months)**

**ITT analysis!**

**CALCULATE SD FROM INTERQUARTILE RANGE**

**MAX - MIN / 1.35== (33-29=4) == (4/1,35=2.96); SD=2.96

**MAX - MIN / 1.35== (32-29=3) == (3/1,35=2.22; SD=2.22 for controls

esizei 95 30.0 2.96 103 30.0 2.22, all

**Intervention - Tai Chi Chuan (mind-body); Control - excersise as usual (passive)**

**Outcome - Quality of sleep (PSQI), Follow-up post-intervention (3 months)**

**ITT analysis!**

esizei 95 4.11 2.09 103 4.27 2.05, all

**2.3. Combined SMDs to avoid unit-of-analysis error**

**2.3.1. Comment:**

Precautions were taken to overcome unit-of-analysis error and avoid using multiple assessment of the same construct (Higgins et al. 2008). If more than one ES was reported in a given study for the same outcome at a given follow-up point (e.g., for depression assessed by both the Hamilton Depression Rating Scale and Beck Depression Inventory), we averaged ESs to obtain the single outcome measure per intervention at each measurement point (Higgins 2006; Jones & Johnston 2000; Kanji et al. 2006; Peden et al. 2001; Seligman et al. 1999). ESs were also averaged within the trials with multiple interventions of similar nature, i.e. if interventions belonged to the same category (Chiauzzi et al. 2008; Mak et al. 2015). A similar approach was applied to studies with multiple comparisons (Chiauzzi et al. 2008; Rohde et al. 2014; Yang et al. 2014). An exception was the study by Kanji N et al. (Kanji et al. 2006), where two control groups – an attention control and a time control – were included separately as considered to be different in approach and content and, thus, representing active and inactive comparisons, respectively.

**2.3.2. Coding for combining Hedges’g as explained in 2.3.1**

For every study that fulfilled the criteria for the risk of unit-of analysis error (from 2.3.1) we used the STATA command: **metan SMD LOWER_CI UPPER_CI, randomi lcols( first_author_pubyr )**, in order to obtain one effect size per study for the same outcome at a given follow-up point.

The following SMDs were combined:

| **First author’s last name and publication year** | **On what variable SMDs and 95%CIs were combined** | **Details of combining** | **At what follow-up point (months)** |
| --- | --- | --- | --- |
| Chiauzzi E, 2008 | Control groups | Control web-site and no treatment | 5 |
| Franklin J, 2012 | Intervention groups | PAAL and Self-regulation | 12 |
| Franklin J, 2012 | Intervention groups | PAAL and Self-regulation | 18 |
| Higgins D, 2006 | Outcomes | Generalized anxiety disorders (measured with GADQ) and anxiety symptoms (measured with BAI) | 6 |
| Higgins D, 2006 | Outcomes | Generalized anxiety disorders (measured with GADQ) and anxiety symptoms (measured with BAI) | 12 |
| Jones M, 2000 | Outcomes | General coping and Direct coping | 3 |
| Jones M, 2000 | Outcomes | State anxiety and trait anxiety | 3 |
| Kanji N, 2006 (A) | Outcomes | State anxiety and trait anxiety | 6 |
| Kanji N, 2006 (A) | Outcomes | State anxiety and trait anxiety | 12 |
| Kanji N, 2006 (B) | Outcomes | State anxiety and trait anxiety | 6 |
| Kanji N, 2006 (B) | Outcomes | State anxiety and trait anxiety | 12 |
| Mak W, 2015 | Step 1: Outcomes  Step 2: Intervention groups | Step 1: Depression, Anxiety and Stress measured with DASS  Step 2: HAPA and Mindfulness |  |
| Mak W, 2015 | Intervention groups | HAPA and Mindfulness (for Mental well-being) |  |
| Mak W, 2015 | Intervention groups | HAPA and Mindfulness (for Persieved stress) |  |
| Peden A, 2001 | Outcomes | Depressive symptoms (measured with CES-D) and depressive symptoms (measured with BDI) | 6 |
| Peden A, 2001 | Outcomes | Depressive symptoms (measured with CES-D) and depressive symptoms (measured with BDI) | 18 |
| Rohde P, 2014 | Control group | Bibliotherapy and Brochure control | 6 |
| Rohde P, 2014 | Control group | Bibliotherapy and Brochure control | 12 |
| Seligman M, 1999 | Outcomes | Anxiety symptoms (measured with HARS) and anxiety symptoms (measured with BAI) | 12 |
| Seligman M, 1999 | Outcomes | Anxiety symptoms (measured with HARS) and anxiety symptoms (measured with BAI) | 18 |
| Seligman M, 1999 | Outcomes | Anxiety symptoms (measured with HARS) and anxiety symptoms (measured with BAI) | 24 (not used in meta-analysis) |
| Seligman M, 1999 | Outcomes | Anxiety symptoms (measured with HARS) and anxiety symptoms (measured with BAI) | 30 (not used in meta-analysis) |
| Seligman M, 1999 | Outcomes | Anxiety symptoms (measured with HARS) and anxiety symptoms (measured with BAI) | 36 (not used in meta-analysis) |
| Seligman M, 1999 | Outcomes | Anxiety symptoms (measured with HARS) and anxiety symptoms (measured with BAI) | 6 |
| Seligman M, 1999 | Outcomes | Depression symptoms (measured with HDRS) and depression symptoms (measured with BDI) | 12 |
| Seligman M, 1999 | Outcomes | Depression symptoms (measured with HDRS) and depression symptoms (measured with BDI) | 18 |
| Seligman M, 1999 | Outcomes | Depression symptoms (measured with HDRS) and depression symptoms (measured with BDI) | 24 (not used in meta-analysis) |
| Seligman M, 1999 | Outcomes | Depression symptoms (measured with HDRS) and depression symptoms (measured with BDI) | 30 (not used in meta-analysis) |
| Seligman M, 1999 | Outcomes | Depression symptoms (measured with HDRS) and depression symptoms (measured with BDI) | 36 (not used in meta-analysis) |
| Seligman M, 1999 | Outcomes | Depression symptoms (measured with HDRS) and depression symptoms (measured with BDI) | 6 |
| Yang W, 2015 | Control group | Assessment-only and Placebo (for BDI-II) | 3 |
| Yang W, 2015 | Control group | Assessment-only and Placebo (for BDI-II) | 7 |
| Yang W, 2015 | Control group | Assessment-only and Placebo (for STAI-I) | 3 |
| Yang W, 2015 | Control group | Assessment-only and Placebo (for STAI-I) | 7 |

**2.4. Hierarchical outcomes**

To analyze the combined negative and positive outcomes, we applied a hierarchical approach for selecting outcomes from the studies reporting more than one from the same category. The hierarchy was based on descending order of outcome reporting, i.e. from the most often reported to the least often reported. For negative outcomes the hierarchical selection was ordered as: depressive symptoms, anxiety symptoms, stress, psychological distress, self-reported worry, quality of sleep and passive coping. For positive outcomes the order was: self-esteem, academic performance, self-efficacy, self-compassion, mental or subjective well-being, resilience, stress management, approach coping and happiness.

**2.4.1. STATA coding for hierarchical outcomes**

*****Generate hierarchical order for negative outcomes at postintervention follow-up point of 3-6 months*****

keep if (positive_negative==”Negative” & FU_3cat==0)

gen negative_hierarchical =1 if OUTCOME==”Depressive symptoms”

replace negative_hierarchical =2 if OUTCOME==”Anxiety symptoms”

replace negative_hierarchical =3 if OUTCOME==”Stress”

replace negative_hierarchical =4 if OUTCOME==”Psychological distress”

replace negative_hierarchical =5 if OUTCOME==”Self-reported worry”

replace negative_hierarchical =6 if OUTCOME==”Quality of sleep”

replace negative_hierarchical =7 if OUTCOME==”Passive coping”

*****Identify duplicates (several negative outcomes within the study at the given follow-up point) and keep the disease with top hierarchical order (lowest number from above)*****

sort first_author_publyear negative_hierarchical

duplicates tag first_author_publyear, generate(multiple_entry)

ta multiple_entry,m

browse if multiple_entry >= 1

list if multiple_entry==1

duplicates drop

duplicates drop first_author_publyear, force

duplicates list

****Same as above to be repeated for** **negative outcomes at postintervention follow-up point of 7-12 months****

keep if (positive_negative==”Negative” & FU_3cat==1)

*****Same as above to be repeated for negative outcomes at postintervention follow-up point of 13-18 months*****

keep if (positive_negative==”Negative” & FU_3cat==2)

*****Generate hierarchical order for positive outcomes at postintervention follow-up point of 3 months*****

keep if (positive_negative==”POsitive” & FU_3cat==0)

gen positive_hierarchical =1 if OUTCOME==”Self-esteem”

replace positive_hierarchical =2 if OUTCOME==”Academic performance”

replace positive_hierarchical =3 if OUTCOME==”Self-efficacy”

replace positive_hierarchical =4 if OUTCOME==”Self-compassion”

replace positive_hierarchical =5 if (OUTCOME==”Mental well-being” | OUTCOME==”Subjective well-being”

replace positive_hierarchical =6 if OUTCOME==”Resiliance”

replace positive_hierarchical =7 if OUTCOME==”Stress management”

replace positive_hierarchical =8 if OUTCOME==”Approach coping”

replace positive_hierarchical =7 if OUTCOME==”Happiness rating”

*****Identify duplicates (several positive outcomes within the study at the given follow-up point) and keep the disease with top hierarchical order (lowest number from above)*****

sort first_author_publyear positive_hierarchical

duplicates tag first_author_publyear, generate(multiple_entry)

ta multiple_entry,m

browse if multiple_entry >= 1

list if multiple_entry==1

duplicates drop

duplicates drop first_author_publyear, force

duplicates list

*****Same as above to be repeated for positive outcomes at postintervention follow-up point of 7-12 months:*****

keep if (positive_positive==”Positive” & FU_3cat==1)

*****Same as above to be repeated for positive outcomes at postintervention follow-up point of 13-18 months:*****

keep if (positive_positive==”Positive” & FU_3cat==2)
