# Supplementary figures and images for: Effects of mental health interventions for students in higher education are sustainable over time: a systematic review and meta-analysis of randomized controlled trials
Source: PeerJ. 2018 Apr 2;6:e4598. doi: 10.7717/peerj.4598 (PMC5885977; doi:10.7717/peerj.4598)

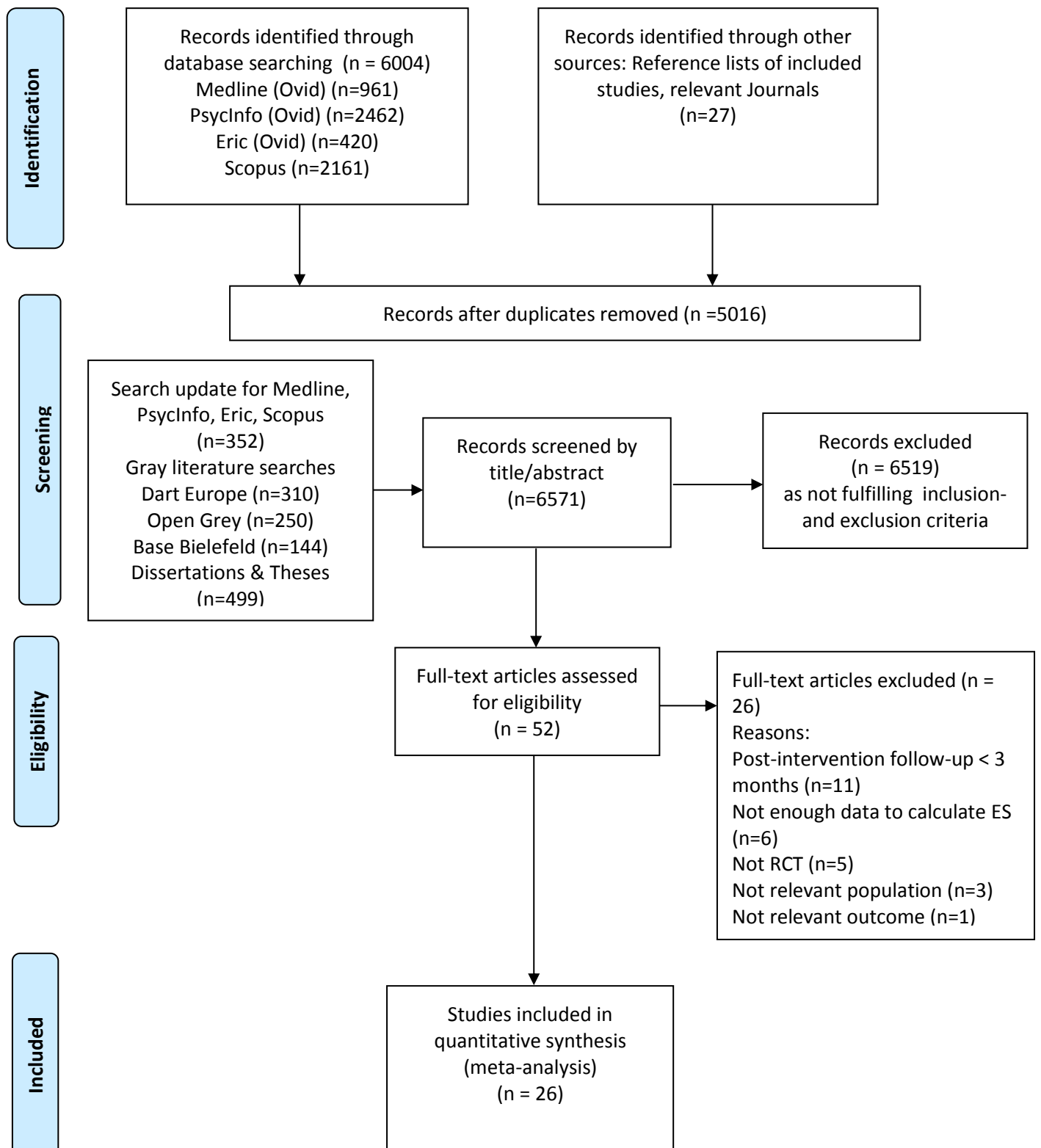

Supplement: Supplemental Information 12 [file peerj-06-4598-s012.pdf]
